# Balancing metabolic optimization and reproductive safety in Polycystic Ovary Syndrome: a Bayesian-informed framework for GLP-1 receptor agonists

**DOI:** 10.3389/fnut.2026.1809416

**Published:** 2026-06-02

**Authors:** Enxue Song, Yuan Fang, Xilian Li, Wenlan Fu, Haiwei He, Zhiyuan Gao, Xue Tian, Biao Gao

**Affiliations:** 1 Department of Obstetrics and Gynecology, The Second Affiliated Hospital of Anhui Medical University, Hefei, Anhui, China; 2 Obstetrics & Gynecology Hospital of Fudan University, Shanghai Key Lab of Reproduction and Development, Shanghai Key Lab of Female Reproductive Endocrine Related Diseases, Shanghai, China; 3 Department of Obstetrics and Gynecology, The Third People’s Hospital of Hubei Province, Wuhan, China; 4 Department of Urology, Changhai Hospital, Naval Medical University, Shanghai, China; 5 Yueyang Integrated Traditional Chinese and Western Medicine Hospital, Shanghai University of TCM, Shanghai, China; 6 Department of Nutrition, Cangzhou Central Hospital, Cangzhou, Hebei, China; 7 Teaching and Research Support Center, Naval Medical University, Shanghai, China

**Keywords:** Bayesian network meta-analysis, fertility management, GLP-1 receptor agonists, Polycystic Ovary Syndrome, preconception care, reproductive safety, washout period, weight loss

## Abstract

**Background:**

GLP-1 receptor agonists (GLP-1RAs) address key metabolic drivers of Polycystic Ovary Syndrome (PCOS), yet their integration into fertility management remains challenging because reproductive efficacy is variably reported, preconception washout is required, and standardized clinical pathways are lacking.

**Main body:**

We present a structured, fertility-centered, hypothesis-generating narrative synthesis of the metabolic–reproductive–pregnancy continuum in PCOS, integrating evidence from randomized trials, observational studies, reviews, and clinical guidance. Heterogeneous findings are organized by intervention/comparator, follow-up window, and key effect modifiers, including baseline obesity/insulin-resistance phenotype, concomitant metformin, and lifestyle co-interventions. The clinical framework is derived from this narrative synthesis rather than from quantitative reproductive meta-analysis. As a methodological proof-of-concept only, we additionally performed a Bayesian network meta-analysis restricted to body-weight change within a prespecified 12–16-weeks window in the largest connected treatment network. Because ovulation, clinical pregnancy, time to pregnancy, and live birth were inconsistently defined and sparsely reported across trials, no confirmatory quantitative synthesis or treatment ranking was undertaken for these reproductive outcomes. We therefore distinguish evidence domains explicitly, with relatively robust support for short-term metabolic benefit but substantially greater uncertainty for reproductive translation and periconception safety.

**Conclusion:**

The narrative evidence supports positioning GLP-1RAs as time-limited, preconception metabolic-optimization tools, not as confirmed fertility-enhancing therapies *per se*. Their use in PCOS fertility care should be individualized through shared decision-making, with explicit discussion of the difference between established metabolic benefit and hypothesis-generating reproductive benefit, together with drug-specific washout planning and strategies to minimize post-discontinuation rebound. Future trials should standardize reproductive endpoints and systematically capture periconception exposure to enable confirmatory synthesis and individualized benefit–risk estimation.

## Graphical Abstract

**Figure:**
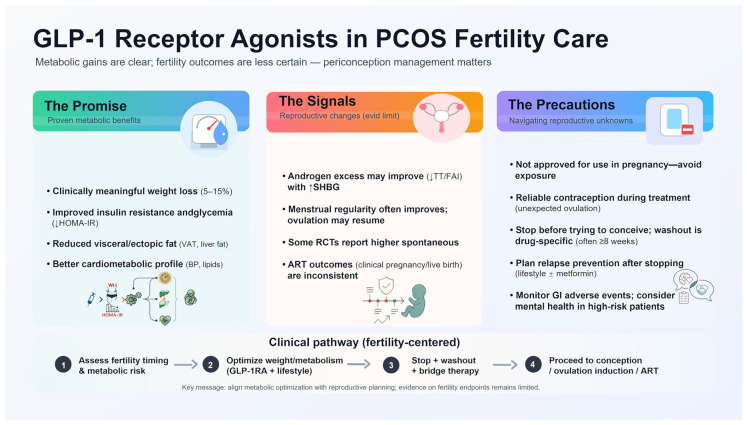
This graphical abstract summarizes the proposed fertility-centered framework for GLP-1 receptor agonist use in PCOS, highlighting metabolic optimization, reproductive timing, preconception washout, and individualized benefit-risk decision-making.

## Highlights

- Clinical integration gap: GLP-1 receptor agonists address the metabolic roots of PCOS, yet fertility-centered pathways remain inconsistent due to limited reproductive endpoint data and periconception safety concerns.
- Evidence synthesis: We integrate RCT evidence on weight and metabolic outcomes with exploratory findings on reproductive outcomes, highlighting key effect modifiers (baseline obesity/IR phenotype, concomitant metformin, and follow-up window).
- Decision framework: We propose a Bayesian- informed, fertility-centered framework that translates heterogeneous evidence into clinically interpretable probabilities to support shared decision-making.
- Safety and timing: We outline practical preconception considerations (treatment duration, washout planning, and transition strategies) and prioritize evidence gaps for future trials.

## 1 Background: metabolic-reproductive challenges of PCOS across lifespan

### 1.1 Epidemiology and reproductive impact

Polycystic Ovary Syndrome (PCOS) is recognized not only as a reproductive disorder in women of childbearing age but also as a complex, lifelong cardiometabolic risk state (1–3). Affecting 6%–13% of women globally, latest reports from the World Health Organization (WHO) indicate that approximately 70% of cases remain undiagnosed, suggesting that the true global burden of the disease is severely underestimated (4–6).

Due to oligo-ovulation or amenorrhea, PCOS leads to a decline in natural conception rates (7–9). Furthermore, the risks of gestational diabetes, hypertension, preeclampsia, and fetal intrauterine growth restriction increased by approximately 1.5–2 times (5, 10, 11). PCOS is also associated with insulin resistance (IR), type 2 diabetes (T2D), metabolic syndrome, metabolic dysfunction-associated steatotic liver disease (MASLD), and atherosclerotic cardiovascular disease (12–16). Consequently, PCOS must be viewed as a lifelong cardiometabolic high-risk state requiring continuous assessment (17).

This risk, superimposed with delayed childbearing, amplifies reproductive uncertainty. Accumulated obesity, IR, and hyperandrogenism potentially damage ovarian reserve, oocyte quality, and endometrial receptivity; meanwhile, advancing age itself brings declines in natural fertility and increased risks of chromosomal abnormalities and pregnancy complications (8, 17). Under the pressure of “dual clocks” (reproductive age and metabolic age), the central challenge in management is balancing metabolic control with pregnancy safety within a finite fertility window.

### 1.2 Central role of obesity and IR in PCOS infertility

Against the backdrop of the “obesity epidemic” and the trend of delayed childbearing, the predominantly obese PCOS phenotype presents compounding challenges to female fertility. Women with PCOS exhibit higher levels of visceral adiposity and IR compared to control populations; notably, the prevalence of IR remains significant even within the lean PCOS phenotype (18–20).

Driven by the IR–hyperinsulinemia–hyperandrogenism axis, metabolic imbalance directly disrupts follicular development, leading to chronic ovulatory dysfunction and deteriorating both oocyte quality and endometrial receptivity (21, 22). The establishment of this pathological mechanism has undergone extensive exploration: from the initial report by Burghen et al. (21) regarding the correlation between hyperinsulinemia and hyperandrogenism, to the foundational work by Dunaif and Nestler (1980–1990) that established the causal relationship between IR and PCOS (23, 24), and through to the debates on the universality of this axis following the formulation of the Rotterdam diagnostic criteria (2003) (25, 26). Ultimately, international evidence-based guidelines have confirmed these pathological drivers of PCOS (17, 27–29). It is now widely accepted that, regardless of the diagnostic criteria applied, all patients should be assessed for metabolic risk.

Insulin exerts a synergistic effect with gonadotropins on both granulosa and theca cells: hyperinsulinemia amplifies luteinizing hormone (LH) signaling and upregulates the expression of steroidogenic enzymes, thereby promoting ovarian and adrenal androgen synthesis. Simultaneously, it suppresses the hepatic synthesis of sex hormone-binding globulin (SHBG), further elevating free testosterone levels (8, 13, 20). This cascade leads to follicular arrest, manifest as the characteristic polycystic ovarian morphology, and causes anovulation or oligo-ovulation. Furthermore, the hyperandrogenic state interferes with endometrial receptivity and impairs embryonic implantation; this is exacerbated by the secretion of leptin, adiponectin, and various inflammatory cytokines from adipose tissue, which participate in the remodeling of the ovarian and endometrial microenvironments, leading to a further decline in oocyte quality and luteal function (13, 18, 20).

Obesity not only impairs natural ovulation and conception but also significantly alters ovarian responses in assisted reproductive technology (ART). Obese patients with PCOS often exhibit increased gonadotropin requirements, a lower yield of retrieved oocytes, a reduced proportion of high-quality embryos, and an elevated risk of ovarian hyperstimulation syndrome (OHSS) during ovulation induction. Additionally, rates of fresh cycle cancellation are higher, leading to compromised cumulative live birth rates (30–32). Simultaneously, obesity and IR amplify the risk of complications by increasing the likelihood of gestational diabetes mellitus and pregnancy-induced hypertension (33, 34). Body weight serves as both an etiological driver and a key therapeutic target. Research indicates that a 5%–10% reduction in body weight is sufficient to significantly improve insulin sensitivity, reduce androgen levels, and restore ovulatory function in a subset of patients (19, 20, 31). However, in real-world clinical practice, relying solely on lifestyle interventions often fails to achieve sustained and clinically meaningful weight-loss goals, thereby creating a clear role for pharmacological assistance in the management of PCOS-related infertility.

### 1.3 The established role of GLP-1 receptor agonists in obesity and T2D

Glucagon-like peptide-1 receptor agonists (GLP-1RAs) were originally introduced as therapeutic agents for T2D. In recent years, they have progressively evolved into pivotal medications for the management of obesity and the provision of cardiorenal protection. Canonical GLP-1RAs, such as liraglutide, semaglutide, and tirzepatide (which possesses dual GLP-1/GIP receptor agonist activity), have consistently demonstrated superior metabolic and cardiorenal outcome benefits in large-scale randomized controlled trials (RCTs) (35–38). These findings have fundamentally reshaped the treatment paradigms for both obesity and T2D.

Regarding weight reduction, pooled results from clinical trials indicate that, compared to placebo, liraglutide at 3.0 mg/d achieves a placebo-subtracted weight loss of approximately 5%. Weekly semaglutide at 2.4 mg can achieve weight reductions of 10%–12%, while tirzepatide has even demonstrated marked improvement in specific trials, reaching 5%–20%+ (35, 38, 39). These effects far exceed those of most lifestyle intervention studies, leading to a rapid improvement in clinical outcomes with GLP-1RAs in obesity management.

More importantly, cardiovascular and renal outcome studies have demonstrated that GLP-1RAs and related multi-receptor agonists not only lower body weight and blood glucose but also significantly reduce the risk of major adverse cardiovascular events (MACE) and the worsening of renal function. The SELECT trial and subsequent renal subgroup analyses suggested that, in populations with both obesity and pre-existing cardiovascular disease, semaglutide not only reduced the risk of cardiovascular events but also slowed the decline of the estimated glomerular filtration rate (eGFR) (40). Compared with traditional GLP-1RAs, tirzepatide has been shown to reduce the incidence of all-cause mortality, cardiovascular events, and adverse renal events in patients with T2D (41, 42).

As indications have expanded from diabetes to obesity, GLP-1RAs have increasingly entered routine care for women of reproductive age, including those with PCOS, in whom obesity and insulin resistance are highly prevalent (36, 38, 43). This expanding use has raised interest in whether these agents can improve the metabolic background preceding conception and thereby support restoration of ovulation or enhance fertility treatment readiness. However, in contrast to the robust metabolic and cardiorenal evidence base, evidence regarding reproductive outcomes, periconception safety, and offspring health remains limited and heterogeneous. Although registry studies and cohort analyses of early pregnancy exposure are available, they remain insufficient to support a definitive conclusion of safety (44–48).

### 1.4 Rationalizing “fertility-centered” review of GLP-1RAs in PCOS: bridging metabolic-reproductive divide

#### 1.4.1 Clinical paradox: tension between metabolic optimization and reproductive urgency

While GLP-1RAs have been established as a first-line intervention for managing metabolic comorbidities in PCOS, their potential teratogenic risks and the requirement for strict preconception washout periods present a formidable clinical dilemma for reproductive specialists. In clinical practice, this conflict manifests as a “time-benefit” trade-off: Is the “reproductive delay” necessitated by a 3–6 months treatment phase and a subsequent 1–2 months washout period justified by the potential improvements in oocyte quality and endometrial receptivity? This delay is particularly critical for patients with advanced maternal age (AMA) or diminished ovarian reserve (DOR), for whom any postponement may result in an irreversible decline in reproductive potential. Furthermore, the recently highlighted “Ozempic babies” phenomenon (unintended pregnancies) has exposed a significant blind spot in current management models: metabolic correction often precedes a patient’s awareness of their recovered fertility, leading to a risk of fetal exposure during the critical “window” of the drug’s washout period.

#### 1.4.2 Evidence gap: metabolic proxies as insufficient substitutes for reproductive outcomes

Despite the proliferation of guidelines and reviews regarding GLP-1RAs, the majority remain focused on metabolic syndrome, weight management, and cardiorenal protection. Existing reproductive medicine literature often relies on theoretical extrapolations–such as the assumption that “weight loss inherently restores ovulation”–but lacks systematic evaluations of hard reproductive endpoints, including natural pregnancy rates, time to pregnancy (TTP), and cumulative live birth rates in ART (8, 19). This disconnect between evidence and practice is most pronounced in the periconception period. Although early pregnancy registries have not identified a stable teratogenic pattern, current guidelines recommend strict precautionary washout periods (44–48), current guidelines typically adopt a defensive posture, recommending strict washout periods prior to planned conception (17, 43). The tension between widespread “off-label” use and these defensive guidelines leaves clinicians without a robust evidence base when facing real-world decision-making challenges.

#### 1.4.3 Real-world decision dilemmas

The friction between prevalent clinical use and existing defensive guidelines is particularly salient in the following three scenarios:

Value assessment of pretreatment before ART: in obese PCOS patients with IR or MASLD, GLP-1RAs are frequently utilized as “weight-loss accelerators” before starting stimulation cycles. However, it remains unclear whether the gains in ovarian responsiveness and cumulative live birth rates achieved through weight loss are sufficient to offset the risks and treatment delays associated with the mandatory washout period.

Controversy over first-line positioning: For obese women seeking to restore natural ovulation, should GLP-1RAs move beyond traditional lifestyle interventions to become a first- or second-line therapeutic option? The optimal timing of intervention and duration of therapy remain undecided.

The void in periconception safety counseling: as the accessibility of these medications increases, it is becoming increasingly common for patients to experience unintended pregnancies before realizing their ovulatory function has improved. Conducting precise teratogenic risk assessments and providing prenatal counseling in the face of early-pregnancy drug exposure remains a daunting challenge for reproductive specialists due to limited evidence.

#### 1.4.4 The scope and strategy of this review

Current metabolic-centric approaches fail to address the reproductive complexities of PCOS. Here, we establish a fertility-centered evaluative framework. By looking beyond simple weight-loss metrics, we systematically integrate evidence concerning metabolism, reproduction, and periconception safety to address the clinical pain points identified above. Specifically, this paper will: (i) Synthesize Evidence: Provide a descriptive analysis of the real-world impact of GLP-1RAs on ovulation, natural pregnancy, and ART outcomes; (ii) Construct Pathways: Offer actionable, evidence-based recommendations for individualized dosing timelines, contraceptive strategies, and washout protocols; and (iii) Explore Methodology: Introduce a Bayesian probability model as a proof-of-concept (PoC) to quantify the posterior probabilities of weight-loss outcomes and infer their potential value in improving reproductive prognosis. Through this multidimensional synthesis, we aim to provide a therapeutic strategy more closely aligned with the complexities of real-world decision-making in gynecological and reproductive medicine.

### 1.5 Literature and methodology

**FIGURE 1:**
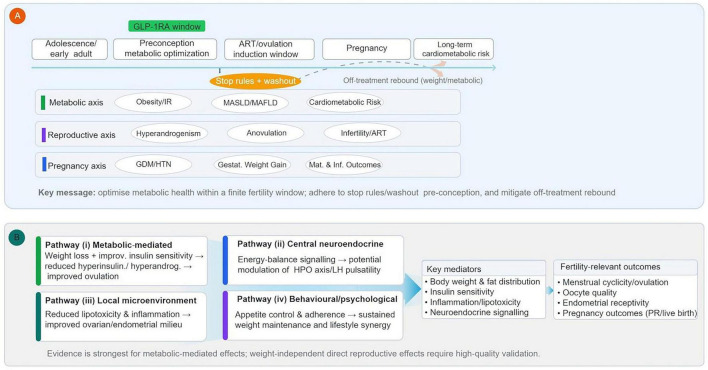
Clinical decision windows and mechanistic framework for integrating GLP-1 receptor agonists into fertility-centered PCOS care. **(A)** The metabolic–reproductive–pregnancy continuum in PCOS across the reproductive life course, highlighting preconception metabolic optimization, the washout window, and the risk of post-discontinuation rebound. **(B)** Our putative pathways by which GLP-1RAs may influence reproductive outcomes: metabolic improvement, central neuroendocrine modulation, microenvironmental remodeling, and behavioral support. These may converge on ovulatory recovery, oocyte competence, endometrial receptivity, and pregnancy potential. Evidence is strongest for the metabolism-driven pathway; direct reproductive effects independent of weight loss remain unconfirmed. Unless otherwise stated, mechanistic pathways discussed below derive from preclinical, translational, or indirect human data and should not be interpreted as established clinical mediators of fertility benefit. PCOS, Polycystic Ovary Syndrome; GLP-1RA, GLP-1 receptor agonist; IR, insulin resistance; MASLD, metabolic-associated fatty liver disease; HPO, hypothalamic–pituitary–ovarian axis; LH, luteinizing hormone.

This review uses a structured narrative evidence synthesis as its primary method of evidence integration. We searched PubMed (up to December 12, 2025) and supplemented this with forward and backward citation tracking to identify pivotal randomized trials, observational studies, systematic reviews, and clinical guidance relevant to GLP-1RA use in PCOS. Because the aim of the review is clinical decision support rather than a formal all-endpoint meta-analysis, priority was given to studies most informative for metabolic outcomes, reproductive endpoints, and periconception management. To improve transparency, an adapted PRISMA-style flow diagram documenting identification, screening, eligibility assessment, de-duplication, and selection of the RCT evidence base and the proof-of-concept Bayesian subset is provided in the Supplementary Appendix Figure 1, together with details of the search strategy, screening process, and data extraction. We additionally summarize the evidence by domain and certainty level in a structured Supplementary Table 6, explicitly separating relatively robust short-term metabolic evidence from more exploratory reproductive and periconception safety findings. To orient readers to the clinical scope of the review, Figure 1 summarizes the fertility-centered decision landscape across the metabolic–reproductive–pregnancy continuum (Panel A). Panel B presents the hypothesis-level mechanistic framework used only to organize subsequent discussion, not to imply confirmed causal mediation.

Rather than attempting confirmatory quantitative synthesis across all outcomes, we deliberately restricted quantitative analysis to a methodological demonstration. Specifically, we performed a proof-of-concept Bayesian network meta-analysis of body-weight change only, within a prespecified 12–16-weeks window, using the largest connected treatment network. The primary model was a random-effects consistency model, with a fixed-effect model used for sensitivity analysis. Weakly informative priors were used; posterior sampling was performed by Markov chain Monte Carlo; and convergence, model fit, and arm-level deviance were assessed in the Supplementary materials. Because the connected network was sparse and only weakly looped, formal inconsistency testing was not treated as decisive, and SUCRA/rank probabilities are interpreted as exploratory summaries rather than confirmatory treatment ordering. Reproductive outcomes, including ovulation, clinical pregnancy, time to pregnancy, and live birth, were not quantitatively synthesized because their definitions, denominators, and follow-up windows varied substantially across studies and event counts were limited. Accordingly, the fertility-centered framework proposed here should be interpreted as hypothesis-generating and narrative-based, with the Bayesian NMA serving only to illustrate how probability-based outputs may assist clinical interpretation of short-term metabolic benefit.

## 2 Pathophysiological basis: coupling of metabolic imbalance and reproductive axis

### 2.1 Heterogeneous phenotypes and neuroendocrine characteristics of PCOS

Polycystic Ovary Syndrome is a clinically heterogeneous syndrome, and diagnosis remains syndromic: clinicians identify combinations of hyperandrogenism, ovulatory dysfunction, and polycystic ovarian morphology (PCOM), while excluding disorders with similar presentations (49). The major diagnostic frameworks (Rotterdam, NIH, and AE-PCOS) rely on overlapping but non-identical feature sets, which can combine metabolically distinct patients within the same cohort (17). For example, the Rotterdam criteria permit a phenotype defined by ovulatory dysfunction and PCOM in the absence of hyperandrogenism, whereas the NIH criteria require hyperandrogenism together with ovulatory dysfunction. As a result, metabolic risk (e.g., adiposity, IR, and related cardiometabolic features) can vary substantially across–and even within–studies using different criteria (49, 50).

Data-driven clustering studies suggest that conventional diagnostic labels can mask biologically coherent subgroups. Across analyses, a recurring contrast is observed between (i) a “reproductive” subtype–often reported as higher LH and SHBG with elevated androgens despite comparatively lower BMI and fasting insulin–and (ii) a “metabolic” subtype, characterized by higher adiposity with higher glucose/insulin measures and lower SHBG (and, in some reports, lower LH) (51). In a larger unsupervised clustering analysis (*n* = 11,908), four sub-phenotypes were identified, broadly echoing this metabolic–reproductive axis and showing substantial overlap with NIH-based subtyping frameworks (52), consistent with prior NIH subtype classifications and related work (50, 53). From a mechanistic perspective, recognizing a metabolic subtype is clinically informative because it frames reproductive dysfunction as potentially downstream of systemic metabolic stress acting on the hypothalamic–pituitary–ovarian (HPO) axis, thereby prioritizing metabolic risk reduction as a plausible lever for improving reproductive function and for tailoring treatment intensity.

From a reproductive endocrine perspective, “canonical” PCOS is characterized by: (i) hyperandrogenism, presenting as hirsutism, acne, and androgenetic alopecia, with elevated total/free testosterone, an increased free androgen index (FAI), and occasionally elevated dehydroepiandrosterone sulfate (DHEAS); (ii) an elevated ratio, where some patients exhibit significantly increased alongside normal or slightly decreased, which promotes follicular recruitment but hinders progression to the dominant follicle stage; and (iii) altered ovarian morphology, involving increased follicle counts and ovarian volume (PCOM), though these findings must be interpreted with caution in adolescent and young adult populations.

The “weighting” of metabolic and reproductive risks varies across phenotypes. The metabolic subtype is more prone to obesity, IR, dyslipidemia, and, making it the primary target population for intervention in this review. Conversely, while the metabolic burden in the reproductive subtype is relatively lighter, ovulatory dysfunction and hyperandrogenic symptoms are more prominent, suggesting that the mode of benefit may differ in these patients (51, 54).

### 2.2 Obesity and IR–hyperandrogenism axis: pathophysiological hub

In PCOS, obesity–particularly visceral adiposity–initiates a complex pathophysiological cascade. Increased levels of free fatty acids and pro-inflammatory cytokines synergistically trigger tissue-level desensitization to insulin (53, 55–60). Attributed to the “selective IR” paradox, compensatory hyperinsulinemia can amplify hyperandrogenism via several mechanisms: (i) cross-activation of insulin and/or IGF-1 receptors, which enhances LH-mediated expression of key steroidogenic enzymes (e.g., CYP17A1) in ovarian theca cells and increases androgen synthesis; (ii) suppression of hepatic SHBG production, thereby increasing free testosterone; and (iii) stimulation of adrenal androgen production (e.g., androstenedione and DHEAS), further augmenting the systemic androgen load (13, 61).

The visceral fat–inflammation–lipotoxicity axis further alters the ovarian microenvironment. Infiltration of M1-type macrophages into visceral fat leads to chronic release of pro-inflammatory cytokines, such as IL-1β, which drive ovarian interstitial fibrosis and hemodynamic changes (62, 63). Meanwhile, the ectopic deposition of in the ovaries and liver directly impairs oocyte mitochondrial function and energy metabolism, resulting in the so-called “lipotoxic oocyte” phenotype (64, 65). This is considered a core mechanism underlying impaired oocyte quality in cycles.

Metabolic dysfunction-associated steatotic liver disease is another prevalent metabolic comorbidity in obese women with PCOS. Even after adjusting for, PCOS remains an independent risk factor for hepatic steatosis and (66), in turn, is associated with an increased risk of gestational diabetes, pregnancy-induced hypertension, and preeclampsia, serving as a critical indicator for preconception and periconception management (67, 68).

### 2.3 GLP-1: from glucose-lowering strategy to a multi-system metabolic protection platform

As a core incretin secreted by enteroendocrine L-cells, not only regulates insulin and glucagon secretion in a glucose-dependent manner but also serves as a central controller of energy homeostasis. It activates receptors on pancreatic cells to promote insulin secretion and inhibit glucagon release; simultaneously, it slows gastric emptying and enhances satiety, thereby reducing postprandial glycemic fluctuations (69, 70). Receptors are expressed in multiple nuclei of the hypothalamus and brainstem, where central mechanisms suppress appetite and regulate energy intake and expenditure (71–74).

Due to the short half-life of endogenous, clinical applications rely on structurally modified. Currently, have evolved from “antidiabetic drugs” into a “platform-type” therapy covering obesity, cardiovascular, and renal protection (75–77). Their pharmacological actions include: (i) weight reduction, achieved by modulating and neuronal activity in the arcuate nucleus and lateral hypothalamus to reduce hunger and rewards-driven eating; delayed gastric emptying further reduces total caloric intake; (ii) improvement in insulin sensitivity, where and indices improve through the combined effects of weight loss and adipose tissue redistribution (78); (iii) optimization of lipid profiles and hepatic fat, reducing triglycerides and non- levels and decreasing hepatic and ectopic fat deposition, thereby improving (79, 80); and (iv) cardiorenal protection, which has led to their inclusion in recommended treatment sequences for obesity and T2D (76, 81, 82).

In PCOS, theoretically creates a more favorable metabolic environment for ovarian function, endometrial receptivity, and pregnancy outcomes through these multi-system actions. This potential reproductive efficacy explains the recent emphasis on clinical research and guidelines (54, 83, 84).

### 2.4 Metabolic–reproductive– psychological axis: multifaceted pathways of GLP-1RA action

Building on Figure 1B, we discuss four non-mutually exclusive routes by which GLP-1RAs may contribute to reproductive improvement in PCOS: (i) metabolic mediation, (ii) central neuroendocrine modulation, (iii) attenuation of lipotoxicity/inflammation, and (iv) psychological and behavioral facilitation. These mechanisms may interact at the ovary and endometrium, with downstream implications for oocyte competence, endometrial receptivity, and pregnancy outcomes.

#### 2.4.1 Indirect metabolic pathway: weight/IR improvement → androgen relief → ovulation

This route is supported by the most consistent clinical evidence. Weight loss and improved insulin sensitivity reduce hyperinsulinemia and increase SHBG, thereby lowering free androgen exposure and decreasing ovarian and adrenal androgen production. Reduced local lipotoxicity and inflammatory signaling may, in turn, favor follicular selection and progression toward dominance, with higher ovulatory frequency (78, 85). Prospective studies further suggest weight reduction is associated with improved menstrual regularity, a more favorable endometrial milieu, and higher rates of ovulation restoration (39, 86–89).

#### 2.4.2 Neuroendocrine–reproductive axis: coupling of appetitive centers and reproductive neurons

The expression of receptors in hypothalamic reproductive nuclei provides a biological basis for a direct influence on the reproductive axis. In animal models, activation of these receptors has been shown to modulate neuronal firing patterns and the KNDy (kisspeptin/neurokinin B/dynorphin) network, thereby influencing GnRH/LH pulsatility and estrous cyclicity (89–91). In women with PCOS, disordered appetite and weight regulation often coexist with menstrual dysfunction; integrating energy and reproductive signals may help restore the physiological “threshold sensing” of adequate energy reserves to support pregnancy. However, clinical evidence demonstrating that reshaping the axis independently of weight change remains scarce.

#### 2.4.3 Local ovarian and endometrial microenvironments: inflammation, lipid metabolism, and receptivity

In animal models of PCOS, attenuating local ovarian inflammation and oxidative stress, improving follicular structure and increasing ovulation counts (83, 92). *In vitro* studies suggest that may enhance endometrial receptivity by improving insulin signaling and inflammatory status in endometrial cells, thereby upregulating the expression of implantation-related molecules such as integrins (84, 86, 92). However, this evidence remains preclinical, and human data is insufficient to distinguish between the direct and indirect effects of on the ovaries and endometrium.

#### 2.4.4 Psychological–behavioral pathway: alleviation of weight stigma and lifestyle remodeling

Women with PCOS frequently experience depression, anxiety, body image distress (related to obesity and hirsutism), and disordered eating tendencies, often rooted in past experiences of failed weight loss (93–95). By facilitating rapid and significant weight loss, provide psychological corrective experiences regarding body image and weight-control efficacy. This, in turn, enhances self-efficacy and long-term adherence to exercise and dietary interventions, creating a positive feedback loop that indirectly benefits reproductive outcomes.

## 3 Metabolic outcomes of GLP-1RAs in PCOS: from evidence synthesis to Bayesian probability integration

The interpretation of clinical evidence presented below follows the core tension between “metabolic risk × fertility window,” as illustrated in Figure 1A: achieving metabolic optimization within a finite reproductive window while strictly adhering to discontinuation and washout protocols to mitigate periconception exposure risks.

### 3.1 Clinical trial landscape: observations on effect size and heterogeneity

Currently, RCTs of GLP-1RAs in PCOS are generally characterized by small sample sizes, single-center designs, and relatively short follow-up periods. However, both the quantity and methodological quality of these studies are increasing. While body weight and metabolic phenotypes remain the primary endpoints, the outcome spectrum is progressively extending toward lipotoxic load [e.g., hepatic fat and visceral adipose tissue (VAT)], inflammatory/cardiometabolic risk markers, and reproductive outcomes with higher clinical decision-making value, such as menstruation, ovulation, and pregnancy. Our focus is not merely on whether an effect is “statistically significant,” but rather on the magnitude of the effect size, the direction of cross-study fluctuations, and the underlying heterogeneity–factors that directly determine comparability and interpretative boundaries.

#### 3.1.1 Liraglutide: weight, ectopic fat, and reproductive endocrine outcomes

In placebo-controlled trials with approximately 26 weeks of follow-up, liraglutide 1.8 mg/d demonstrated stable, clinically meaningful weight loss, accompanied by improvements in several reproductive endocrine markers. Nylander et al. reported that, compared to placebo, liraglutide significantly reduced body weight (by approximately 5 kg) and increased the proportion of regular menstrual bleeding; concurrently, SHBG levels rose, free testosterone decreased, and ovarian volume showed a directional decrease. However, changes in the reproductive axis and ovarian morphology were not consistent across all endpoints, and gastrointestinal adverse events were common (96, 97).

Regarding lipotoxicity, Frøssing et al. demonstrated that liraglutide significantly reduced hepatic fat content and VAT, resulting in a marked decrease in NAFLD prevalence. Meanwhile, increases in SHBG and decreases in free testosterone showed borderline significance. These findings suggest that the “weight loss–alleviation of lipotoxicity–improvement of hyperandrogenism” cascade exhibits strong consistency at the RCT level, although IR markers (e.g., HOMA-IR) do not necessarily improve synchronously (79).

Evidence for a higher dose (3.0 mg/d) comes from Elkind-Hirsch et al., who observed further increases in weight loss and a decline in the FAI. However, gastrointestinal reactions also increased accordingly, suggesting a practical trade-off between dose-response and tolerability. This must be evaluated within the broader clinical context of “metabolic benefit–fertility window–discontinuation risk management” (98).

In terms of combination strategies, a series of open-label studies by Jensterle-Sever et al. indicated that for patients with a poor prior weight-loss response to metformin (MET), the MET + GLP-1RA combination might yield more substantial weight and metabolic improvements, with safety profiles characterized primarily by transient gastrointestinal reactions (99–101). Notably, as these studies were largely open-label with varying intensities of lifestyle intervention and control conditions, they are better suited as evidence for “strategic feasibility” rather than as direct equivalents to placebo-controlled trials.

As the literature moves from metabolic endpoints to reproductive outcomes, evidentiary certainty declines substantially. In the pilot IVF pretreatment trial by Salamun et al., metformin plus low-dose liraglutide yielded higher clinical pregnancy rates per embryo transfer and higher 12-months cumulative pregnancy rates despite similar weight loss between groups; however, the study enrolled only 28 women, used an open-label design, and did not include validated intermediate measures of endometrial receptivity or other mechanistic correlates. These findings should therefore be interpreted as exploratory and hypothesis-generating rather than confirmatory evidence of a direct reproductive effect independent of weight reduction (102). Similar caution applies to more recent short-term combination studies reporting improvements in ovulation, endocrine markers, or related reproductive parameters, because endpoint definitions, denominators, concomitant therapies, and follow-up windows varied across trials (103, 104).

#### 3.1.2 Semaglutide: mechanistic endpoints and “effect amplification” in combination therapy

Evidence for semaglutide tends to focus on mechanistic and tissue-distribution endpoints. Using nuclear medicine techniques, Jensterle et al. showed that semaglutide delays gastric emptying (prolonging the half-emptying time), providing a direct physiological basis for its effects on appetite and reduced intake (105). Another MRI study suggested it reduces tongue fat deposition, which correlates with changes in weight and waist circumference; however, this endpoint serves more as a mechanistic clue than a direct clinical benefit (106).

Within combination therapy frameworks closer to clinical management, Chen et al. combined semaglutide with MET and observed greater improvements in weight and multiple metabolic markers, along with differences in natural pregnancy rates during the follow-up (107). However, the interpretation of these outcomes is complex, as they involve the confounding effects of drug combination, extended follow-up, and changes in conception-related behaviors. The evidence level and inferential boundaries for such results should be clearly distinguished from those of short-term, placebo-controlled trials.

#### 3.1.3 Dulaglutide: marginal gains within the context of lifestyle intervention

The key finding regarding dulaglutide is that the incremental benefit of the drug diminishes when the control group is already engaged in a structured calorie-restricted diet (CRD). Zhang et al. compared dulaglutide + CRD with CRD alone and suggested potential advantages in certain fat distribution and metabolic risk markers. However, no consistent inter-group differences were observed in reproductive outcomes, such as menstrual frequency or hormonal profiles, and adverse effects were primarily gastrointestinal (108). Thus, this evidence supports the conclusion that, in addition to rigorous lifestyle interventions, pharmacological gains must be evaluated via stratification based on specific populations and target endpoints.

#### 3.1.4 Exenatide and its combination with MET: from metabolic improvement to pregnancy outcomes

The evidence chain for exenatide is relatively long, encompassing early RCTs focused on menstruation, ovulation, and metabolism, as well as follow-up studies targeting pregnancy outcomes. Elkind-Hirsch et al., in a 24-weeks intervention, indicated that exenatide monotherapy or its combination with MET was overall superior to MET monotherapy in terms of weight, abdominal fat, and insulin sensitivity. This was accompanied by improvements in menstrual frequency and ovulation markers, suggesting weight loss and the alleviation of central obesity are likely critical mediators of reproductive benefit (109).

In sub-populations with pre-diabetes, Tao et al. reported that combination therapy was more favorable for glycemic outcomes and included a post-discontinuation observation period to reflect real-world preconception risk management. However, the comparability of such studies depends heavily on the definition of washout periods, follow-up windows, and pregnancy criteria (110). Liu et al. and Li et al. extended observations to pregnancy, noting higher natural pregnancy rates in the short term, though differences in long-term total pregnancy rates and outcomes were less stable. Regression analyses suggested that natural pregnancy was associated with weight loss and improvement in IR, indicating that the path from “metabolic improvement to reproductive benefit” is more likely driven by individual metabolic responses rather than by drug class alone (111, 112).

Various combination therapies and omics studies suggest that GLP-1RAs may influence the metabolic-endocrine network through lipid and bile acid pathways. These findings should be positioned as “hypothesis-generating,” and their clinical translatability requires validation in larger samples with pre-specified endpoints (113–116). Nevertheless, it is clear combination therapies are superior or at least non-inferior regarding weight, BMI, waist circumference, and certain glycemic markers, with safety profiles dominated by mild-to-moderate gastrointestinal reactions.

Summary: Heterogeneity is not merely noise but an “explainable” component of the data. The most consistent and comparable endpoints remain weight loss and the associated reduction in lipotoxic load. In contrast, reproductive outcomes–such as hormonal profiles, ovulation, and pregnancy–show greater cross-study fluctuation. The primary drivers of this variance include: (i) differences in control types (placebo *vs.* MET *vs.* intensive lifestyle); (ii) the presence or absence of concomitant MET or other metabolic agents; (iii) variations in baseline phenotypes (BMI, degree of IR, infertility status/entry into ART pathways); (iv) inconsistencies in follow-up windows and washout protocols; and (v) differences in blinding and reporting standards. Therefore, for subsequent analyses to provide actionable clinical probabilities, it is essential to select endpoints with consistent definitions and stable network connectivity (e.g., weight) and discuss their effect sizes and uncertainties within a standardized time window.

### 3.2 Systematic reviews and meta-analyses: three tiers of critical evidence

Existing secondary research can be broadly categorized into three tiers: (i) PCOS-specific meta-analyses of RCTs (primarily focusing on weight, metabolism, and select endocrine markers); (ii) systematic reviews centered on GLP-1RA monotherapy or combination therapy (emphasizing treatment strategies and “mediating phenotypes” such as body composition and visceral fat); and (iii) guideline-oriented evidence syntheses within the broader “anti-obesity medication spectrum” (focusing on relative effects and evidence gaps). These three tiers are generally consistent: “certainty of benefit” is concentrated in phenotypes related to body weight and central obesity, whereas conclusions regarding IR and reproductive outcomes are more dependent on study design and patient phenotype. In this context, heterogeneity should not be dismissed as mere statistical noise.

The first tier, exemplified by De Hollanda et al., included four RCTs (85) and indicated that GLP-1RAs reduce BMI, waist circumference, triglycerides, and total testosterone. However, no stable differences were observed for HOMA-IR or total cholesterol, suggesting that while benefits for “weight, lipid metabolism, and androgens” are relatively consistent, the “IR endpoint” may be more heavily influenced by sample size, follow-up duration, and baseline IR severity. Lin et al. expanded the number of RCTs (78) and found that the direction of effect for weight/BMI remained consistent, with an overall improvement in IR markers, albeit alongside an increased incidence of adverse events such as nausea, vomiting, and dizziness. This indicates that safety signals are more “visible” in pooled short-term RCTs, while long-term adherence and the risks of weight rebound or metabolic withdrawal post-discontinuation remain difficult to quantify reliably in existing studies.

The second tier, represented by Bader et al. (117), emphasizes that regardless of monotherapy or combination regimens, reductions in BMI, waist circumference, fat mass, and visceral fat mass are consistent across most studies, with combination therapies often showing a greater magnitude of change. However, significant variations in control types (placebo, MET, or intensive lifestyle), concomitant medications, and follow-up windows mean that a robust head-to-head ranking of strategies is still lacking.

The third tier, typified by Goldberg et al. (118), primarily serves as a framework for evidence-based guidelines. Their conclusions similarly highlight the advantages of GLP-1RAs for weight and select metabolic/clinical phenotypes but explicitly note that the evidence chain for long-term follow-up, lean PCOS, and reproductive endpoints (ovulation, pregnancy, and live birth) remains weak. Furthermore, periconception safety and discontinuation management have yet to be standardized in study designs.

Overall, existing secondary research provides a relatively solid, yet imperfect, evidence base for the metabolic benefits of GLP-1RAs in PCOS. Their “combinability” for meta-analysis is limited by structural factors: comparators, time windows, enrollment phenotypes, and washout periods. Consequently, the analysis of outcomes does not rely primarily on binary “significant/non-significant” conclusions. Instead, we anchor our analysis to the most consistent and network-stable outcome–body weight–which serves as the most reliable proxy for subsequent reproductive benefit translation. We observe effect magnitudes and fluctuation structures within a unified context to provide an actionable evidence interface for washout management and sequential therapy in the fertility pathway.

### 3.3 Weight management and body composition optimization: “foundational effect” for reproductive benefit

Across the diverse landscape of evidence involving various comparators and time windows, weight loss and improvements in body composition emerge as the most consistent and clinically interpretable benefit findings of GLP-1RAs in PCOS, particularly within obesity- and IR-dominant phenotypes. For patients planning natural conception or preparing for ART, “how much weight is lost” is merely the starting point; the critical factors are “when the target is achieved” and whether a relatively stable weight and metabolic state can be maintained during the discontinuation washout period. These factors determine whether metabolic gains can be effectively translated into reproductive success. Accordingly, this section explores the magnitudes of effects, clinical thresholds, and body composition characteristics to establish a foundation for subsequent discussions of IR, lipotoxicity, and reproductive outcomes.

Meta-analyses suggest that GLP-1RAs can lead to an average BMI reduction of 1–2 kg/m^2^, a decrease in waist circumference of several centimeters, and total weight loss of 5%–7% in some studies (85). It is important to note that the apparent magnitude of the weight-loss effect is often influenced by baseline levels, control conditions, and follow-up windows; therefore, it is more appropriate to express clinical significance in terms of “probability of achieving a threshold” rather than a single mean difference. One study on semaglutide reported an average 6-months weight loss of approximately 11.5 kg (roughly 10%–15% of baseline weight) and observed a high proportion of improvement in menstrual cycles (39). Given that such data are not entirely homogenous with RCTs in terms of design and control conditions, they are best utilized as exploratory indicators of “potential effect ceilings and accessibility” to calibrate clinical expectations, rather than as direct points of comparison for placebo-controlled RCTs.

In clinical practice, weight losses of ≥5% and ≥10% are commonly used thresholds for “clinical significance.” PCOS guidelines state that a weight reduction of ≥5% can significantly improve insulin sensitivity and hyperandrogenic load, while ∼10% weight loss is strongly linked to restored ovulation and improved pregnancy rates (17). Within the 3–6 months treatment window typical of existing RCTs, a substantial proportion of participants achieved the 5% threshold. While the ≥10% threshold is attainable in some small-sample studies, these estimates are highly sensitive to covariates and should not be generalized as a universally reproducible proportion (39, 78).

At the level of body composition, limited DEXA and CT evidence suggests that GLP-1RAs primarily reduce fat mass–specifically visceral fat–while relatively preserving lean mass (54, 75). This is particularly critical for women of reproductive age: the former is more closely associated with alleviating chronic low-grade inflammation, while the latter’s loss would be detrimental to pregnancy and labor endurance and could compromise the stability of metabolic and nutritional reserves within the ART pathway.

It must be emphasized that this “foundational effect” is not merely about a change in the numbers on a scale; it represents a fundamental remodeling of the metabolic environment. This includes attenuating the “amplification effect” of hyperinsulinemia on the ovaries, reducing lipotoxic and inflammatory loads, and potentially creating a more favorable endometrial metabolic microenvironment. Thus, weight and body composition optimization provide a more predictable physiological state for subsequent reproductive interventions, the success of which hinges on improving the IR hub and its heterogeneous structure.

### 3.4 Glucose–lipid metabolism and IR

Insulin resistance serves as the crucial hub connecting metabolic derangements and reproductive dysfunction in PCOS, making it a primary target for GLP-1RA intervention within the “metabolic–reproductive” axis. Meta-analyses indicate that GLP-1RAs generally reduce HOMA-IR and improve fasting blood glucose and 2 h OGTT levels in the PCOS population (78). Although a single RCT failed to observe a statistically significant improvement in HOMA-IR (85), this discrepancy is more likely attributable to limited sample sizes, restricted follow-up windows, and heterogeneity in baseline metabolic phenotypes rather than a lack of efficacy. A consistent pattern emerges in phenotypes with a high metabolic burden, such as obesity and IGT or pre-diabetes, improvements in fasting glucose, OGTT curves, and HbA1c are more pronounced, with some subjects even reverting from IGT to normoglycemia. Conversely, in lean PCOS or those with a lighter metabolic load, GLP-1RAs typically yield smaller reductions, as baseline glucose and HOMA-IR levels are often within the normal range. In these cases, the clinical significance of intervention must be weighed against absolute risk and specific therapeutic goals (54, 118).

The value of improving IR extends far beyond glycemic index optimization. Reducing hyperinsulinemia attenuates its stimulatory effect on ovarian steroidogenesis, thereby alleviating the hyperandrogenic load at its source. Furthermore, enhancing the endometrial metabolic microenvironment and glucose transport may provide a more favorable foundation for early embryonic development and implantation. By mitigating metabolic risks before conception, GLP-1RAs may also indirectly reduce the incidence of future gestational complications, such as gestational diabetes mellitus (GDM). However, few prospective studies have conducted prespecified, systematic causal pathway analyses linking these metabolic improvements to reproductive endpoints such as ovulation, clinical pregnancy, or live birth rates. The translational chain from “metabolic benefit to reproductive success” remains a critical gap that high-quality research must address. The following section validates IR improvement through organ-level “metabolic phenotypes,” specifically focusing on ectopic fat load, represented by MASLD/hepatic fat and VAT.

### 3.5 Lipid profile, MASLD, and proactive intervention strategies

Beyond IR, PCOS is frequently characterized by an atherogenic dyslipidemia profile (e.g., elevated triglycerides, decreased HDL-C, and increased non-HDL-C). Even after adjusting for age and BMI, this lipid profile remains associated with an increased cardiovascular risk (11, 53). Concurrently, the risk of MASLD is approximately 2–2.5 times higher in the PCOS population–a correlation that persists after adjusting for age and BMI (66, 119)–positioning MASLD as the “hepatic phenotype” of systemic IR. Crucially, MASLD is not merely a passive marker of metabolic derangement; longitudinal cohort studies suggest it is linked to adverse periconception outcomes, including GDM, preeclampsia, and fetal growth restriction. These may form a detrimental intergenerational cycle via the “maternal metabolic disturbance–placental function–fetal growth” pathway (68, 120–122).

Regarding lipid and hepatic endpoints, GLP-1RAs act through mechanisms distinct from those of traditional lipid-lowering agents. Meta-analyses suggest that they reduce triglycerides and waist circumference, although their effects on total cholesterol and LDL-C are inconsistent (85). This indicates that their cardiometabolic benefits are primarily mediated by weight loss, IR alleviation, and improvements in triglyceride-rich lipoprotein pathways, rather than a direct reduction in LDL-C levels. Of greater clinical translational significance, RCTs have demonstrated that approximately 26 weeks of liraglutide treatment can significantly reduce hepatic fat content and improve transaminase levels, outperforming lifestyle interventions alone (79). Furthermore, when transitioned to metformin maintenance post-discontinuation, some patients exhibit limited weight regain and maintain lower body weight and hepatic fat levels compared to baseline (123).

From the perspective of the “preconception–pregnancy” continuum, the presence of MASLD indicates that women with PCOS are on a high-risk trajectory for both gestational complications and long-term adverse cardiometabolic outcomes (66, 121). Although prospective studies specifically targeting “PCOS + MASLD” with pregnancy outcomes as the primary endpoint are currently lacking, a robust inference can be drawn based on the pathophysiological framework of MASLD and evidence from the general population: proactively identifying and reducing MASLD and ectopic fat load prior to pregnancy may be a key step in improving both periconception outcomes and long-term prognosis (68, 76). Since GLP-1RAs simultaneously target IR, lipid profiles, and hepatic fat, they provide an actionable pharmacological tool for “risk-forward” preventive strategies (78, 83).

### 3.6 Clinical decision-making: Bayesian probability using weight as an exemplar

To address the limitations of binary evidence (i.e., focusing solely on “significant vs. non-significant”) in clinical decision-making and to provide more intuitive data for individualized counseling, we conducted an exploratory Bayesian NMA, intended as a PoC, which introduces the ROPE to translate heterogeneous clinical evidence into posterior probabilities of achieving or exceeding predefined clinical thresholds.

#### 3.6.1 Defining ROPE and clinical significance

While frequentist meta-analyses are widely applied within the PCOS evidence-based framework, their conclusions often fail to directly address threshold-based clinical questions. To enhance the interpretability of the evidence, we utilized body weight (kg) as the exemplar endpoint for this analysis.

We standardized the outcomes as “incremental weight loss relative to metformin” (Δ weight loss, kg; where Δ > 0 indicates greater weight loss than metformin and Δ < 0 indicates less). The ROPE was defined as the interval where the difference is considered clinically negligible: | Δ| ≤ δ. We set the primary threshold at δ = 1.0 kg (with δ = 0.5 kg for sensitivity analysis) to derive two categories of communicable probabilities: (i) Pr(| Δ| ≤ δ), the posterior probability of falling within the ROPE, indicating that the difference compared to the control is clinically negligible (approximate equivalence); and (ii) Pr(Δ > δ) [or Pr(Δ < -δ) where applicable], the posterior probability that the advantage (or disadvantage) exceeds the clinical threshold, quantifying the likelihood of “clinically substantial incremental benefit (or harm).”

It is important to emphasize that while the ≥5%/≥10% thresholds discussed in Section “3.3 Weight management and body composition optimization: “foundational effect” for reproductive benefit” refer to “absolute weight loss” for an individual, the δ (kg) used in this PoC-NMA expresses the “probability of incremental benefit relative to a control,” and thus the two represent distinct evaluative metrics.

#### 3.6.2 Exemplary analysis: posterior probability synthesis of metabolic benefits

**FIGURE 2:**
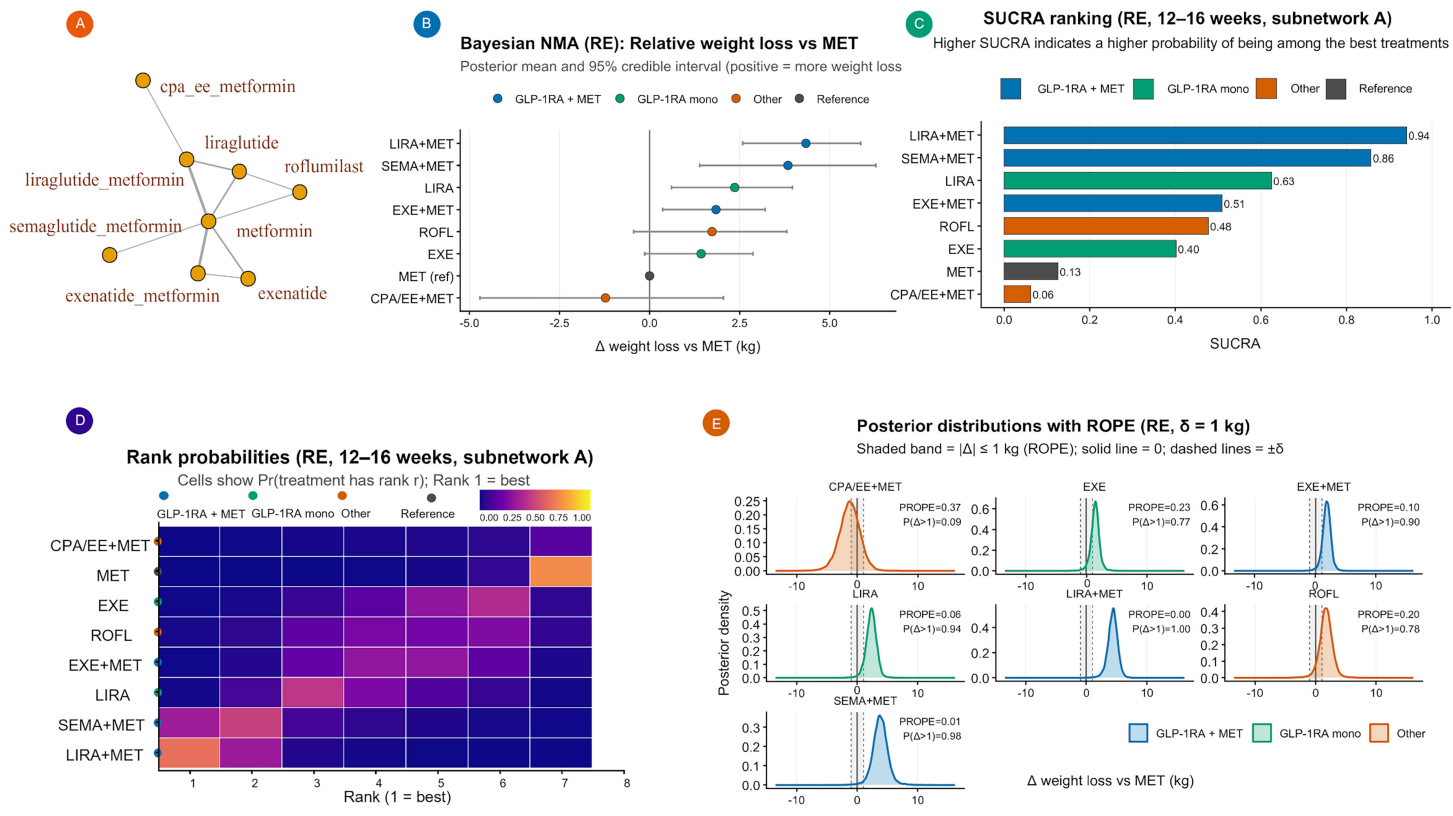
Bayesian consistency NMA of weight loss in the primary short-term window (12–16 weeks, maximum connected subnetwork). **(A)** (Network geometry). Node size is proportional to the cumulative sample size; line thickness reflects the number of direct comparisons. **(B)** [Relative effects *vs.* Metformin (RE Model)]. Points denote posterior means; horizontal lines represent 95% CrI. Positive values (Δ > 0) indicate greater weight loss than metformin. **(C)** (SUCRA rankings). Higher values (0–1) indicate a higher cumulative probability of being the most effective treatment. Colors denote treatment categories. **(D)** (Rank probability heatmap). Treatments are ordered by SUCRA. Darker cells indicate a higher probability of achieving a specific rank [*Pr*(*rank* = *r*)]. **(E)** (Posterior densities with ROPE, δ > 1 kg). Curves show the posterior distribution of weight loss differences relative to metformin. The gray shaded region represents the region of practical equivalence (ROPE, |Δ|≥1 kg). Annotations indicate the probability of equivalence (*P*_*ROPE*_) and clinically meaningful advantage [*P*(Δ > 1kg)]. Because this proof-of-concept network was sparse, limited to the largest connected 12–16-weeks subnetwork, and only weakly looped, consistency was evaluated pragmatically through model comparison and fit diagnostics rather than relying on formal node-splitting as a decisive test; accordingly, **(C,D)** (SUCRA and rank probabilities) should be interpreted as exploratory summaries of relative ordering rather than confirmatory treatment ranking.

Focusing on body-weight change within the prespecified 12–16-weeks window, we analyzed the largest connected treatment subnetwork, comprising 11 trials, 8 treatment nodes, and 25 study arms (Figure 2A and Supplementary Table 1). The primary analysis used a random-effects consistency model, with a fixed-effect sensitivity analysis. Because the network was sparse and only weakly looped, formal node-splitting/local inconsistency analyses were not considered sufficiently informative to serve as a primary decision tool. Instead, consistency was judged pragmatically through clinical transitivity, inspection of network geometry, comparison of random-effects and fixed-effect results, and deviance-based model fit diagnostics. Full Bayesian model specification, convergence diagnostics, fit statistics, and supportive consistency considerations are provided in the Supplementary Appendices 1–6; Supplementary Figures 1–3, Supplementary Tables 2–5.

The random-effects relative treatment estimates versus metformin are shown in Figure 2B, followed by exploratory SUCRA and rank-probability summaries in Figures 2C,D and posterior density/ROPE summaries in Figure 2E. Relative to metformin, liraglutide plus metformin and semaglutide plus metformin showed very high posterior probabilities of achieving more than 1.0 kg additional weight loss, while liraglutide monotherapy and exenatide plus metformin also showed high posterior probabilities of clinically meaningful benefit. By contrast, exenatide and roflumilast were associated with greater uncertainty, with 95% credible intervals crossing the null. Tightening the ROPE threshold to 0.5 kg did not materially change the overall interpretation (Figure 2E and Supplementary Table 5). SUCRA values and rank probabilities placed liraglutide plus metformin, semaglutide plus metformin, and liraglutide monotherapy near the top of the exploratory ordering (Figures 2C, D), but these rankings should be interpreted as descriptive rather than confirmatory in a sparse proof-of-concept network. The fixed-effect sensitivity analysis preserved the overall directional ordering, supporting the robustness of the main interpretation (Supplementary Appendix 6).

#### 3.6.3 Uncertainty management and the clinical pathway

Beyond aggregate effects, the Bayesian framework offers a more intuitive representation of the potential range of “individual response” through posterior predictive distributions, effectively transforming heterogeneity from a “statistical artifact” into a tool for managing clinical uncertainty. For metabolic endpoints, the relatively concentrated distributions imply that efficacy is highly predictable, supporting “certainty-based selection” in clinical practice. Conversely, when this framework is extended to reproductive endpoints–which are subject to greater definitional heterogeneity and effect modification–the appearance of long-tailed distributions suggests that the quantified probability of efficacy may not demonstrably exceed that of metformin. This provides a theoretical basis for uncertainty management, serving as a crucial caveat for clinicians: when utilizing GLP-1RAs, one must distinguish between the relative certainty of metabolic benefits and the stochastic nature of reproductive improvements. Such an approach optimizes expectation management and supports more prudent, individualized decisions regarding combination therapies. Probabilistic inference and strategic ranking should only be undertaken once outcome definitions, time windows, and network geometry have reached a state of stability. In essence, Bayesian outputs do not offer “additional” conclusions; rather, they transform metabolic gains into communicable, stratified, and “foundational” decision-making information.

### 3.7 Summary of metabolic outcomes: building foundation for fertility treatment

Across available trials, GLP-1RAs consistently improve metabolic drivers that are clinically relevant to fertility planning in PCOS. Several patterns emerge. First, weight loss is a reproducible finding over a 3–6-months window, and in many cohorts the mean reduction reaches ranges commonly considered clinically meaningful for reproductive planning (often cited around ≥5% of baseline weight), with some studies reporting larger decreases. Second, improvements in IR and glycaemic indices are most apparent in higher-burden phenotypes, particularly in women with impaired glucose tolerance/prediabetes or related metabolic comorbidities, where changes in HOMA-IR, fasting glucose and OGTT profiles are more pronounced and may plausibly reduce periconception metabolic risk. Third, reductions in ectopic and visceral adiposity appear clinically relevant. Randomized data and follow-up reports indicate decreases in hepatic fat surrogates and waist-based measures, suggesting improvement in the metabolic milieu entering pregnancy. Fourth, evidence synthesis would benefit from complementing mean differences with threshold-based metrics. For fertility decision-making, average effects alone often do not map cleanly onto “how likely this patient is to achieve a clinically meaningful change.” Bayesian hierarchical approaches can express benefits as posterior probabilities around pre-specified thresholds, which may better support stratified counseling and shared decisions.

These metabolic findings set the stage for the next clinical questions that matter in fertility care: (i) whether any reproductive benefits (ovulation or pregnancy outcomes) occur beyond weight loss; (ii) when along the preconception pathway treatment should be initiated; and (iii) how discontinuation should be timed, including washout and bridging strategies. Addressing these questions requires linking metabolic gains to the hyperandrogenism–ovulation–pregnancy axis and explicitly managing initiation and washout within the patient’s fertility timeline.

## 4 Hyperandrogenism, ovulatory function, and fertility outcomes: a reproductive perspective

### 4.1 Remodeling of the androgen profile and endocrine homeostasis

Hyperandrogenism is a hallmark phenotype of PCOS, serving as the link between metabolic derangement and ovulatory dysfunction via the “insulin–ovary–hepatic SHBG” axis. Consistent with the metabolic improvements discussed previously, current evidence supports the premise that GLP-1RAs promote SHBG synthesis and reduce bioavailable free androgen activity primarily through weight loss and the alleviation of IR. Whether GLP-1RAs exert direct modulation of the hypothalamic-pituitary-ovarian (HPO) axis beyond these metabolic pathways remains to be verified by clinical mechanistic evidence.

#### 4.1.1 Evidence and potential mechanisms

GLP-1 receptor agonists demonstrate a statistical advantage in improving androgen-related markers. Compared with lifestyle interventions or metformin, they significantly elevate SHBG and modestly reduce total testosterone (with a standardized mean difference, SMD approx. −0.39), although the direction and magnitude of effects on FAI and DHEAS exhibit heterogeneity (124). These endocrine benefits are more pronounced in overweight or obese phenotypes; studies have observed that when treatment duration exceeds 20 weeks, and weight loss exceeds 5%, recovery of SHBG and resolution of “functional hyperandrogenism” are more pronounced and correlate with both baseline HOMA-IR and the magnitude of weight loss (125, 126). Studies involving exenatide have also reported its superiority over metformin in reducing total testosterone and FAI, with clearer signals observed in populations at higher metabolic risk (118, 127).

#### 4.1.2 Limitations and research bottlenecks

A major challenge in current research is the difficulty of “decoupling” the mediating effect of weight loss from the drugs’ direct pharmacological actions. Most trials have not reported outcomes stratified by weight-loss achievement, nor have they incorporated mediatory analysis designs involving insulin clamps, hepatic fat content, or visceral adiposity. Consequently, a more prudent conclusion is that the improvements in the androgen profile observed with GLP-1RAs are primarily secondary endocrine effects following metabolic optimization. Direct modulation of the HPO axis remains a hypothesis that requires further validation through mechanistic trials.

### 4.2 Challenges in menstrual regularity reconstruction and ovulation restoration

Improved menstrual regularity is often regarded as a visible indicator of therapeutic success. Existing studies generally suggest that, by alleviating the “obesity/IR–hyperandrogenism” cascade, GLP-1RAs bring the HPO axis closer to a physiological state.

#### 4.2.1 Clinical and morphological benefits

Meta-analyses indicate a statistical advantage for GLP-1RA groups in “menstrual regularity improvement” (e.g., SMD = 1.72) (124). Specific studies on exenatide have also reported superior menstrual regularity compared to metformin after 12–24 weeks of treatment (128, 129). Concurrently, imaging data suggest that liraglutide treatment leads to reductions in ovarian volume and antral follicle count (AFC), indicating that ovarian morphology can undergo measurable changes as the metabolic-endocrine environment improves (96, 124, 130).

#### 4.2.2 The “gray area” of evidence: menstruation ≠ ovulation

It must be emphasized that improved menstrual frequency does not equate to the restoration of ovulation. First, most studies utilize “menstrual bleeding” as a proxy for ovulation rather than mid-luteal progesterone, serial ultrasonography, or other gold-standard confirmations. Second, the definition of “regular menstruation” varies across studies (e.g., cycle length, frequency, or scoring scales), and limited sample sizes and follow-up durations make it difficult to exclude the impact of anovulatory bleeding. Thus, it is currently more appropriate to state that while GLP-1RAs improve oligomenorrhea and suggest a potential recovery of ovulatory function, the evidence for a definitive “increase in ovulation rate” remains weak. High-quality trials with standardized ovulation monitoring are urgently needed.

### 4.3 Natural pregnancy and TTP: evidence for a “window effect”

In this section, reproductive outcomes are summarized descriptively rather than quantitatively synthesized *de novo*, because available studies differ substantially in endpoint definition, denominators, post-treatment conception windows, and washout structure. From a reproductive perspective, the currently available evidence is concentrated mainly on the natural conception pathway and is most consistent with the possibility that metabolic optimization may improve the conception window and shorten time to pregnancy (TTP) in selected women with PCOS.

#### 4.3.1 Available evidence for natural pregnancy rates and TTP

Published meta-analytic summaries and follow-up data from individual trials suggest that GLP-1RA-based metabolic optimization may be associated with higher natural conception rates and shorter time to pregnancy in selected overweight or obese women with PCOS (111, 112, 124). However, these data should be interpreted cautiously. Across studies, definitions of pregnancy-related endpoints, treatment duration, discontinuation structure, washout timing, concomitant metformin use, and post-treatment conception attempts vary substantially. Moreover, the available reports do not allow confident separation of direct drug-related reproductive effects from mediation through weight loss and improvement in insulin resistance. Therefore, the current evidence is more appropriately interpreted as suggesting a possible conception-window benefit after metabolic optimization, rather than confirming a reproducible treatment effect on hard reproductive outcomes (111, 112, 124).

#### 4.3.2 Methodological limitations and clinical strategies

Existing studies generally treat pregnancy-related outcomes as secondary or exploratory endpoints and seldom report analyses stratified by weight-loss achievement, insulin-resistance improvement, or washout structure (111, 112, 124). As a result, the critical question of whether any observed pregnancy benefit is independent of metabolic change remains unresolved. This limitation is especially important in PCOS, where improved natural conception may plausibly reflect restoration of ovulatory opportunity after weight loss rather than a direct fertility-enhancing effect of the drug itself. In addition, post-treatment conception windows are heterogeneous, and the timing of treatment discontinuation relative to conception attempts is rarely standardized. Accordingly, this evidence should be regarded as low-to-moderate certainty and hypothesis-generating. For current practice, the most defensible use of GLP-1RAs is as a time-bounded metabolic optimization strategy in women with meaningful obesity/insulin-resistance burden, with reproductive endpoints interpreted as potential downstream gains rather than guaranteed direct effects (17, 111, 112, 124).

### 4.4 ART-related outcomes: limited signals, fragmented evidence, and “target population” boundary

In the context of ART, PCOS is often associated with excessive but poor-quality follicles, OHSS risk, and impaired endometrial receptivity. Theoretically, optimizing the metabolic microenvironment could improve ART outcomes, but current evidence is derived from small-scale exploratory studies, requiring caution in extrapolation.

#### 4.4.1 Clinical evidence and limitations

An open-label RCT compared 12 weeks of MET versus MET + low-dose liraglutide (1.2 mg/d) as pretreatment before IVF in obese infertile women (with a 4-weeks washout). While weight loss was similar between groups (approx. 7 kg), the combination group showed significantly higher clinical pregnancy rates per transfer cycle (85.7% vs. 28.6%) and cumulative pregnancy rates over 12 months (102). Although these results hint at potential “beyond-weight” reproductive benefits (83), the extremely small sample size (*n* = 28), open-label design, and lack of objective measurements for key biological intermediates (e.g., oocyte/embryo quality and endometrial receptivity), combined with controversies over the adequacy of the washout period, mean these conclusions should be viewed as exploratory rather than definitive.

#### 4.4.2 Mechanistic hypotheses and clinical positioning

Animal studies suggest that GLP-1RAs may restore ovulation by reducing local ovarian lipotoxicity and inflammatory/oxidative stress loads, thereby improving autophagy and PI3K/AKT/mTOR-related imbalances (131). However, these mechanisms have not been confirmed in humans through a robust “follicular fluid–oocyte–embryo–endometrium” evidence chain. Based on the strength of evidence and safety boundaries, a more rational clinical positioning is to reserve GLP-1RAs as an individualized tool for “intensive metabolic optimization” prior to ART in refractory PCOS individuals with high BMI and prior weight-loss failure. For PCOS patients with normal or low weight, blind extrapolation should be avoided due to the lack of evidence and the higher potential risks associated with further weight loss.

### 4.5 Pregnancy and maternal-fetal outcomes: limited evidence and risk communication priorities

Periconception safety should be framed as an evidence-limited risk-management problem rather than as evidence of safety. To improve transparency, Supplementary Table 7 summarizes molecule-specific pharmacokinetic and clinical considerations, including approximate half-life, label-informed discontinuation or washout recommendations before planned conception, representative human pregnancy-exposure evidence, reported adverse outcomes, and an interpretive certainty judgment. Across agents, the available human pregnancy-exposure data remain sparse, exposure timing is heterogeneous, and existing studies are underpowered for rare outcomes; accordingly, the absence of a stable teratogenic pattern should not be interpreted as proof of safety (44, 45, 47, 132).

From a practical standpoint, the most defensible position remains planned exposure avoidance: explicit fertility planning, reliable contraception during treatment, label-informed discontinuation before conception attempts, immediate cessation once pregnancy is recognized, and active metabolic maintenance during the washout period to reduce rebound-related gestational risk (45, 132–137). This framing is particularly important in PCOS, where restoration of ovulation during treatment may increase the likelihood of unintended conception before patients recognize that fertility has improved.

#### 4.5.1 Post-discontinuation weight rebound and gestational risk

Evidence for post-discontinuation weight rebound and related gestational risk is derived predominantly from non-PCOS or mixed-population cohorts rather than from PCOS-specific longitudinal studies. In these populations, discontinuation of GLP-1RAs before or during early pregnancy has been associated with greater gestational weight gain and, in some reports, less favorable pregnancy-related metabolic outcomes; however, residual confounding by baseline BMI, glycemic status, indication, and discontinuation timing remains difficult to exclude (137). Accordingly, rebound in the periconception period should be viewed as a clinically plausible translational concern rather than as a phenomenon established specifically in women with PCOS. From a practical standpoint, discontinuation strategies should therefore address not only timing, but also the post-cessation stabilization of body weight and metabolic status during the washout period.

#### 4.5.2 Early pregnancy exposure: risk characterization under limited data

Existing registry, cohort, and systematic-review data have not identified a stable pattern of major structural malformations after brief early-pregnancy exposure to GLP-1RAs, but the exposed human evidence base remains limited and heterogeneous. However, this should be interpreted cautiously. The available studies differ in drug type, dose, indication, timing of exposure, comparator structure, and baseline maternal metabolic risk, and the number of exposed pregnancies remains insufficient to exclude uncommon but clinically important harms (44, 47, 132, 133). Thus, the most accurate interpretation is not “safe,” but rather “no stable teratogenic signal identified to date, with evidence still insufficient for a safety conclusion (44, 45, 132).” In clinical counseling, this distinction matters: inadvertent early exposure should generally prompt individualized risk communication and obstetric follow-up, not automatic reassurance and not automatic inference of major harm.

#### 4.5.3 Risk communication: clarifying uncertainty

Current evidence is insufficient to classify GLP-1RAs as “relatively safe” during pregnancy, and they should certainly not be used as a first-line treatment for gestational obesity or diabetes simply due to their weight-loss benefits (45, 47, 132). For women of reproductive age with PCOS, the priority is to simultaneously strengthen fertility planning and contraceptive management while improving ovulatory opportunities, ensuring weight and metabolic stability during the preconception washout period to avoid the maternal-fetal risks associated with a “weight loss–rebound” cycle (133, 137).

### 4.6 Quality of life and psychology: benefits driven by symptom and weight improvement

Quality of life (QoL) impairment in PCOS is frequently linked to weight distress, hirsutism, and infertility-related anxiety. Limited research suggests that approximately 6 months of liraglutide treatment, in addition to achieving weight loss (approx. 9 kg), reduces depression scores and improves QoL dimensions such as mood and self-image, with changes correlating with the magnitude of weight loss (138). At this stage, it is more accurate to describe these psychological and QoL improvements as “concomitant effects” of metabolic and symptomatic relief rather than independent psychopharmacological actions. Future research should utilize PCOS-specific instruments (e.g., PCOSQ) and pre-specify psychological outcomes to enhance comparability and interpretability.

## 5 Safety, clinical tolerability, and periconception management strategies

From the perspective of actionable clinical management, this section categorizes three core challenges associated with the use of GLP-1RAs in women of reproductive age with PCOS: (i) discontinuity in adherence driven by gastrointestinal adverse effects; (ii) identification and monitoring of rare but serious adverse events; and (iii) post-discontinuation weight rebound and its subsequent disruption of the preconception window.

### 5.1 Common adverse effects and adherence gaps

The most prevalent adverse effects of GLP-1RAs are gastrointestinal (GI) symptoms, including nausea, vomiting, and diarrhea or constipation. These typically manifest during the initiation and dose-titration phases and may partially subside over time (77, 139, 140). In PCOS-related trials, even with low starting doses and gradual titration, discontinuation rates of approximately 5%–15% are frequently observed (125, 128).

It is critical to emphasize women of reproductive age may be more sensitive to the subjective burden of nausea and restricted food intake, particularly regarding the interference with social and professional life; real-world studies also suggest higher rates of discontinuation and intermittent use (141, 142). Therefore, clinical success depends on the prerequisite of “long-term persistence.” Implementing target-oriented follow-up, adjusting dietary structures (small, frequent, low-fat meals), and personalizing the titration pace are often more effective in reducing early attrition than a singular focus on weight-loss metrics.

### 5.2 Rare serious adverse events: individualized screening and monitoring

Gallbladder disease: GLP-1RAs are associated with an increased risk of gallbladder-related events, often occurring in parallel with mechanisms such as rapid weight loss and cholestasis (143). Given that PCOS is frequently comorbid with steatotic liver disease and hypertriglyceridemia, we recommend intensified screening for gallbladder symptoms (e.g., right upper quadrant pain, fatty food intolerance) and regular monitoring of hepatobiliary markers during phases of rapid weight loss.

Pancreatitis and Medullary Thyroid Carcinoma (MTC): while existing evidence has not established a definitive causal link, screening for and avoiding use in patients with a history of pancreatitis, MTC, or a family history of Multiple Endocrine Neoplasia type 2 (MEN2) remains a mandatory precautionary measure (144–148).

Ocular risks: recent matched cohort studies have suggested a potential association between semaglutide prescriptions and an increased risk of Non-arteritic Anterior Ischemic Optic Neuropathy (NAION) (Hazard Ratio ≈ 4.28, with inter-study estimates ranging from 4 to 7); however, this remains observational evidence requiring further validation (149). Screening for optic neuropathy history is prudent. Sudden vision loss warrants immediate ophthalmological referral rather than automatic attribution to therapy, as data from large-scale cohorts remain inconsistent.

### 5.3 Adherence and weight rebound in periconception: from “discontinuation node” to “stabilization window”

Weight rebound following discontinuation is a clinically important concern in periconception management. However, the currently available longitudinal evidence on this issue is derived mainly from the general obesity population rather than from PCOS-specific cohorts. In non-PCOS obesity studies, substantial partial regain of prior weight loss after discontinuation of GLP-1RA therapy has been documented, together with attenuation of cardiometabolic benefit (150–152). Whether the magnitude, tempo, and reproductive consequences of rebound are the same in women with PCOS remains unknown. We therefore discuss rebound here as a clinically plausible translational concern, not as a phenomenon established by PCOS-specific prospective evidence.

In PCOS, rebound could plausibly compromise the stability of metabolic and reproductive gains during the preconception interval, particularly where restoration of ovulatory function appears to depend on sustained improvement in adiposity and insulin resistance. However, direct evidence linking post-GLP-1RA rebound to renewed ovulatory dysfunction, shortened fertile-window durability, or worsened pregnancy outcomes in PCOS is still lacking. This should be regarded as a major evidence gap and a priority for future longitudinal studies.

A practical periconception management approach is as follows: (i) Target achievement: Continue treatment alongside lifestyle intervention until pre-specified weight and metabolic targets are met (e.g., 5%–10% weight loss with improvement in IR markers). (ii) Stabilization: Enter a maintenance phase emphasizing dietary structure, resistance training, sleep, and stress management, with the aim of minimizing weight and metabolic fluctuations before conception attempts or ovulation induction. (iii) Discontinuation and washout: For semaglutide, product labeling advises discontinuation at least 2 months before a planned pregnancy (135, 153). More broadly, some reviews have suggested a conservative class-level washout approach of at least 4 weeks where direct evidence is lacking, although this should not replace molecule-specific label guidance (154). (iv) Post-cessation metabolic support: Metformin may be considered during the maintenance phase when clinically indicated. SGLT2 inhibitors may improve metabolic profiles in selected non-pregnant patients, but any such use must remain strictly outside pregnancy, under reliable contraception, and with guideline-consistent discontinuation before conception attempts because of urogenital adverse effects and contraindications during pregnancy (155, 156).

### 5.4 Youth and fertility planning: shifting from “short-term loss” to “cross-cycle net benefit”

Young women with PCOS are often more susceptible to aesthetic pressure and social media influence, potentially viewing GLP-1RAs as a tool for “rapid weight management.” Concurrently, concerns regarding long-term medication and pregnancy safety often lead to intermittent use and frequent “start-stop” cycles.

Stratified management: for those with a BMI ≥ 35 kg/m^2^ and significant metabolic derangement, GLP-1RAs should be integrated as a long-term management option across the reproductive life cycle. For those with a BMI of 25–30 kg/m^2^, the drugs are better positioned as “preconception metabolic optimization tools,” with an emphasis on post-discontinuation maintenance and washout strategies.

Lifestyle as the foundation: the marginal gains from pharmacological intervention depend on the simultaneous reinforcement of lifestyle changes. In the absence of a behavioral foundation, the weight rebound following drug cessation is often more rapid than that following lifestyle intervention alone. Only by reshaping dietary and exercise habits can the vicious cycle of “discontinuation-rebound” be broken.

Proactive fertility planning: parallel assessments of fertility and metabolic risk should be conducted during the early 20s and 30s. Integrating the “intervention window–washout–stable maintenance–conception attempt” sequence into a long-term plan is far more valuable than reactive crisis management after infertility has already manifested.

In the young PCOS population, GLP-1RAs should be conceptualized as a “metabolic-reproductive integrated management tool.” Its net benefit is derived from sustainable metabolic stability and a planned conception window, rather than merely transient weight loss. Future decision-making frameworks based on phenotype stratification and Bayesian integration should prioritize identifying who requires long-term maintenance versus a short-term window, and how to minimize the reproductive costs of post-discontinuation rebound.

## 6 Periconception management and fertility planning: opportunities and risks

In reproductive-aged women with PCOS, the use of GLP-1RAs is best approached as a time-bounded strategy embedded within the fertility plan, rather than a single yes/no decision. When pregnancy is desired, management hinges on (i) explicit reproductive intent, and (ii) reliable contraception until treatment is stopped. A practical sequence is metabolic optimization on therapy → planned discontinuation → label-informed washout → post-discontinuation weight/metabolic stabilization → conception attempt or ART. This approach is designed to meet regulatory expectations and, at the same time, reduce two foreseeable risks: inadvertent early pregnancy exposure and metabolic rebound after cessation.

### 6.1 Regulatory standpoints and guideline consensus

As human safety data are scarce and animal studies suggest reproductive toxicity, regulators currently advise discontinuing GLP-1RAs immediately upon pregnancy confirmation. Washout periods for planned conception remain guided by pharmacokinetics and labeling, in the absence of direct clinical evidence.

Semaglutide: product labeling advises discontinuation at least 2 months before a planned pregnancy, reflecting its long elimination time and extended exposure window (153, 157, 158).

Liraglutide: product information advises that liraglutide should not be used in pregnancy and should be discontinued if pregnancy occurs or is planned, based on limited human data and animal reproductive-toxicity signals (136, 159).

Special warning for tirzepatide: delayed gastric emptying induced by tirzepatide may reduce the exposure and efficacy of oral hormonal contraceptives. Patients are advised to use barrier contraception or switch to non-oral methods during the initiation phase and for 4 weeks following each dose escalation; some labels also suggest a washout window of at least approximately 1 month before attempting conception (160–162).

Guidelines: the 2023 International PCOS Guideline positions GLP-1RAs as practical weight-loss tools for obese women with PCOS but emphasizes that they must be used within a framework of continuous, effective contraception throughout the treatment period, and should not be considered a routine intervention for weight or metabolic management during pregnancy (17).

Lactation: although preliminary data suggest extremely low milk transfer and no clear adverse signals in infants (163), the overall evidence remains insufficient. Mainstream recommendations still favor avoidance or initiating use only after breastfeeding has ceased (132, 164).

### 6.2 Clinical and registry data on pregnancy exposure: relatively “reassuring” but far from “safe”

As prescriptions expand and the preconception window lengthens, accidental periconception exposure has become a practical reality in reproductive clinics. Current evidence generally presents two concurrent perspectives:

Absence of distinct teratogenic signals: aggregated data from registries, cohorts, and systematic reviews indicate that brief exposure to GLP-1RAs in early pregnancy has not been associated with a stable increase in the risk of major structural malformations or miscarriage (44, 165, 166). This supports a clinical approach for “accidental exposure” focused on risk communication and intensified prenatal monitoring rather than mechanical recommendations for termination; however, the sample sizes remain insufficient for a definitive judgment of “absolute safety” (132).

The practical impact of metabolic withdrawal: large-scale population studies suggest that individuals who use and then discontinue GLP-1RAs before pregnancy may experience excessive gestational weight gain (GWG) and an increased risk of gestational diabetes mellitus (GDM) and gestational hypertension (137). Even when baseline metabolic risks are considered as confounders, these results emphasize that periconception management must look beyond the “cessation node” and focus on whether weight and metabolism enter a controllable stability window post-discontinuation.

Accordingly, we suggest that communication should focus on “uncertainty management”–neither exaggerating teratogenic risks nor downplaying the metabolic and pregnancy costs of post-discontinuation rebound.

### 6.3 The “Ozempic babies” phenomenon: fertility recovery vs. contraceptive failure

The “Ozempic babies” phenomenon (unintended pregnancies associated with GLP-1RAs) is consistent with an epidemiological explanation: metabolic improvement triggers the recovery of ovulatory function while contraceptive strategies lag, leading to increased unintended pregnancies. Key mechanisms include the relief of hypothalamic-pituitary-ovarian (HPO) axis inhibition through weight loss and improved insulin sensitivity, alongside improved endometrial receptivity driven by increased SHBG and decreased free androgens (83, 167, 168). Conversely, some patients still adhere to the perception that “long-term infertility equals difficulty in conceiving”; combined with the impact of gastrointestinal reactions on the absorption and adherence of oral contraceptives, the risk is further amplified, particularly during the initiation or dose-escalation phases of tirzepatide (161, 162, 169).

### 6.4 Integrating reliable contraception into the prescription workflow

Therefore, “fertility planning and contraception” should be a mandatory discussion item when initiating GLP-1RAs: patients must be informed of both the potential “restart” of their fertility and the specific circumstances under which oral contraceptive regimens may require adjustment.

Effective contraception is mandatory prerequisite for the use of GLP-1RAs in women of reproductive age with PCOS (17). To reduce the “cognitive-behavioral” disconnect, a structured strategy should be adopted:

Preference for long-acting reversible contraception (LARC): intrauterine devices (IUDs) or subdermal implants are unaffected by delayed gastric emptying and offer superior adherence; these should be the preferred recommendation for obese women with PCOS.

OCP adjustment: for patients who must use oral contraceptive pills (OCPs) for cycle regulation, the risk of reduced efficacy must be clearly communicated if using tirzepatide. Adding barrier contraception during the dose-titration phase is advised, or switching to a vaginal ring or transdermal patch (160, 162).

Pre-prescription triple assessment: (i) fertility intentions (near-term, long-term, or uncertain); (ii) current contraceptive method; and (iii) planned discontinuation and washout schedule. This upgrades the therapeutic goal from “weight loss” to “comprehensive reproductive health management.”

### 6.5 Preconception pathway: “smooth transition” from metabolic optimization to conception

The goal of periconception management is not merely to achieve the lowest possible weight, but to ensure the patient enters a window of relative weight and metabolic stability after an adequate washout before attempting conception. We propose a pathway divided into three sequential stages:

Intensive phase (metabolic optimization): for those planning pregnancy within 6–12 months, GLP-1RAs can be used for 3–6 months of intensive weight loss (using a 5%–10% reduction as an actionable threshold), while simultaneously improving background risks such as IR, MASLD, and lipid profiles. For those with long-term fertility goals, maintenance and relapse prevention should be emphasized.

Transition phase (discontinuation–washout–stabilization): strictly implement clearance times based on drug characteristics–discontinue semaglutide for ≥2 months, tirzepatide for at least 1 month (161), and long-acting formulations like exenatide for at least 3 months (153, 158, 159, 162). This stage is not for “passive waiting” but for establishing anti-rebound mechanisms, including intensified lifestyle interventions. For those with IR, metformin can be prioritized as foundational metabolic management; any “bridging therapy” must be individualized within the boundaries of periconception contraindications (170).

Conception/ART phase: before entering a conception attempt or ART, metabolic stability (weight trajectory, glycemic/IR status, lipids, liver enzymes, etc.) should be re-verified. Clinicians should also monitor nutritional risks associated with rapid weight loss, such as deficiencies in iron, folic acid, and B vitamins, to ensure the patient is “pregnancy-ready” rather than just “weight-ready.” Given that GLP-1RAs improving IVF outcomes remains limited, their appropriate positioning remains a metabolic optimization tool before ART rather than a standardized pretreatment regimen (48).

## 7 From evidence to decision: estimating net benefit under uncertainty

Clinical decisions around GLP-1RAs in fertility-focused PCOS care are rarely binary. They require balancing anticipated metabolic gains against timing constraints, discontinuation requirements, and residual uncertainty in reproductive outcomes. A pragmatic way forward is to (i) use Bayesian inference to express uncertainty in clinically interpretable probabilities, and (ii) use data-driven phenotyping (including ML-enabled clustering where appropriate) to describe heterogeneity in treatment response. These framing centers two practical questions: Which patients are most likely to benefit, and how can treatment be sequenced to maximize net benefit within an individual fertility timeline?

### 7.1 Bayesian perspective: turning evidence asymmetry into communicable probabilities

The evidence base for GLP-1RAs in PCOS is asymmetric. Metabolic endpoints (e.g., weight change, HOMA-IR, HbA1c) are most often supported by RCTs and therefore show relatively consistent and reproducible effects. By contrast, reproductive endpoints (ovulation, natural conception, and live birth) are commonly derived from smaller trials, shorter follow-up periods, or secondary analyses, which yield wider uncertainty intervals and increase susceptibility to imprecision and chance variation. Furthermore, periconception safety and complications rely on registries and retrospective cohorts, where confounding factors cannot be ignored (48, 132). These varying evidence sources possess distinct bias structures: RCTs provide average efficacy under “ideal conditions” but often exclude high-risk or complex patients; real-world studies are more reflective of clinical practice but are prone to indication bias and attrition; and case series or media narratives may capture early concerns or preliminary observations, but are inadequate for quantifying risk.

The value of the Bayesian framework lies not only in its ability to rank evidence but in its logical structure–“Prior + Likelihood → Posterior”–which explicitly maps differences in evidence strength to differences in uncertainty. In practice, this facilitates three actionable inferential postures: (i) Metabolic Endpoints: Characterized by high informational content and concentrated posterior distributions; even with the inclusion of new small-sample studies, the conclusions remain robust. (ii) Reproductive Endpoints: Given the limited information content, weak priors or conservative assumptions are warranted. The resulting posterior intervals often remain wide, suggesting that while benefits are possible, the evidence is insufficient for strong commitments. (iii) Safety Endpoints: Rare events and data scarcity place the posterior in a “gray zone,” best presented through probabilistic language that defines the boundaries of risks that “can neither be confirmed nor excluded” (132).

Consequently, clinical communication can shift from binary assertions (effective/ineffective, safe/unsafe) to a framework of “probabilities + intervals + acceptance thresholds.” For instance, the “posterior probability of reaching a clinically meaningful weight-loss threshold” can be presented alongside the “posterior uncertainty of benefits in hard reproductive endpoints,” rather than replacing the decision process with a single *P*-value.

### 7.2 Decision matrix: unifying multi-endpoint trade-offs via “net utility”

In the PCOS reproductive context, a single pharmacological intervention simultaneously affects metabolic gains, the conception window, and the risk of adverse events. To avoid a fragmented focus on weight or pregnancy rates alone, clinical intuition can be formalized into an Expected Net Utility framework:


$$U\,\left(D\,e\,c\,i\,s\,i\,o\,n\right)=w_{1}\cdot P\,\left(L\,i\,v\,e\,B\,i\,r\,t\,h\right)+w_{2}\cdot P\,\left(M\,e\,t\,a\,b\,o\,l\,i\,c\,B\,e\,n\,e\,f\,i\,t\right)-$$

$$w_{3}\cdot P\,\left(A\,d\,v\,e\,r\,s\,e\,E\,v\,e\,n\,t\right)-w_{4}\cdot C\,o\,s\,t$$

Where *P* represents the probability of key outcomes and *w* represents the patient’s preference weights for those outcomes. The objective of this model is not to calculate a precise decimal value but to deconstruct the decision into three communicable variables: Evidence (probability) – Preferences (weight) – Constraints (risk and cost).

Two typical scenarios illustrate the directional conclusions of this model:

Metabolism-driven: for patients with BMI ≥ 35 kg/m^2^, significant IR, and no near-term fertility plans, *w*_2_ typically dominates. Given the robust metabolic evidence, the net utility is more likely to be positive.

Reproductive-driven: for patients with lower BMI, a primary complaint of infertility, and good ovarian reserve, *w*_1_ is maximal. However, if evidence for hard endpoints (e.g., live birth) is insufficient and costs or washout constraints are significant, the net utility may approach zero or remain uncertain.

### 7.3 From PCOS to “endotypes”: the role of ML in stratification over promises

The diagnostic label of PCOS encompasses a highly heterogeneous pathophysiological spectrum. Unsupervised clustering studies suggest identifiable “endotypes” with distinct reproductive and metabolic characteristics, such as the differentiation between reproductive-dominant and metabolic-dominant subtypes (51, 171, 172). This endotype perspective is critical for GLP-1RA use: if an individual’s primary driver is IR and a lipotoxic background, the posterior probability of response to GLP-1RAs is theoretically higher. Conversely, if the core issue is functional imbalance at the HPO axis level, priority should be given to pathways focused on ovulation induction.

The rational positioning of ML should be to treat “GLP-1RA response” as a new predictive outcome. Based on clinically available variables (BMI, waist circumference, OGTT/HOMA-IR, lipid profiles, NAFLD assessments, androgens, SHBG, and ultrasound markers), interpretable risk stratification models should be established rather than replacing clinical logic with “black-box” algorithms (173, 174). Significant modeling experience has already been accumulated in PCOS screening and ART outcome prediction, with a move toward interpretability assessments such as SHAP values (175–182). In the field of obesity, efforts are underway to use multi-omics and clinical data to predict individual responses to weight-loss interventions (including GLP-1RAs) and to precisely predict the risk of side effects (e.g., nausea) using algorithms like Phenomix GRS. These provide highly valuable methodological benchmarks for the PCOS landscape (183–185).

Notably, usable models must satisfy several criteria: cross-center training and external validation, prospective evaluation, bias and fairness audits, and interpretable presentation. A “high AUC” alone does not equate to a translatable clinical tool.

### 7.4 Multi-modal learning health systems: embedding evidence updates into clinical workflow

Over longer time scales, the application of GLP-1RAs in PCOS lies at the intersection of metabolic intervention and reproductive management. To prevent evidence updates from lagging behind clinical practice, this review proposes a Learning Health System (LHS) objective: iteratively updating risk stratification and decision support through continuous real-world data streams rather than relying on static conclusions.

Input layer: standardized data are paramount. This includes metabolic baselines (BMI/waist circumference, blood pressure, lipids, NAFLD assessment, OGTT/HOMA-IR), reproductive endocrine/ultrasound markers (AMH, AFC, LH/FSH, ovarian volume/morphology), and QoL scales. Where feasible, genetic/omic data (e.g., PRS, metabolomics/lipidomics) can help identify high-responder subgroups (186–188).

Model layer: the modeling strategy prioritizes interpretability and continual updating. Interpretable architecture (or *post hoc* explainers) can identify the main drivers of recommendations (e.g., SHAP-based feature attributions). Bayesian updating can then incorporate emerging RCT and cohort evidence, revising effect estimates and widening or narrowing credible intervals as the evidence base evolves.

Output layer: decision support is framed as a comparison of clinical pathways rather than a single-point prediction. For example, “metabolic optimization with a GLP-1RA first” can be contrasted with “immediate ovulation induction/IVF” with respect to expected trade-offs in live birth, OHSS risk, pregnancy complications, and longer-term metabolic benefits. Presenting these outcomes side-by-side makes it explicit which inferences are supported by robust evidence, and which remain highly uncertain (176, 187, 189).

Overall, the purpose of combining ML and Bayesian components is to improve transparency and updatability, supporting shared decision-making under heterogeneity and incomplete evidence, while avoiding spurious over-precision in reported risks or benefits (173).

## 8 Clinical positioning and pathways: risk-stratified decision matrix

### 8.1 Bridging gap between evidence and guidelines

Traditional stepped care for PCOS-related infertility typically positions lifestyle intervention as the first-line therapy, followed by ovulation induction (OI) with letrozole or clomiphene, and ultimately ART. The clinical integration of GLP-1RAs represents a “pathway advancement” rather than a direct reproductive induction therapy; these agents influence the conception window and ART preparation by remodeling the underlying metabolic substrate. The 2023 International PCOS Guideline remains cautious about the role of GLP-1RAs: their core indications remain limited to weight management and metabolic risk control. Furthermore, the guidelines emphasize that periconception safety evidence remains insufficient, requiring use within a framework of continuous effective contraception, and they do not recommend routine use during pregnancy or lactation (17, 133, 154, 190, 191).

However, practice in reproductive endocrinology reflects a trend where clinical demand has emerged ahead of definitive evidence. Specifically, the use of GLP-1RAs as a “metabolic pretreatment” before IVF/ICSI is increasing, though much of this remains off-label exploration (192, 193). Therefore, we propose a rational conceptual framework: the periconception metabolic optimization tool. Its primary value lies in alleviating IR and lipotoxic load, thereby mitigating the potential negative impact of a detrimental metabolic microenvironment on oocytes and the endometrium. This creates more controllable conditions for subsequent OI or ART, rather than serving as a direct means to “induce ovulation” or “guarantee pregnancy.”

### 8.2 Stratified clinical strategies by phenotype and reproductive intent

As evidence for metabolic benefits currently outweighs that for hard ART endpoints, treatment decisions should be stratified by metabolic risk and the reproductive timeline. We propose a “metabolic risk × reproductive timeline” framework to delineate optimal GLP-1RA use, incorporating specific therapeutic windows and discontinuation protocols.

*Scenario A: “Metabolic Rescue” (High Risk, Suitable for Application)*

Target population: women with BMI ≥ 30 kg/m^2^ plus marked IR or metabolic syndrome; and/or patients with a high metabolic burden who have already entered the fertility pathway but encountered metabolic-related barriers during ART (e.g., high OHSS risk, suboptimal oocyte/embryo quality, or cycle cancelation).

Strategic positioning: positioned as a metabolic foundation reconstruction before ART. The goal is to “reduce uncontrollable metabolic fluctuations to an acceptable range” rather than replacing the ovarian stimulation protocol with a GLP-1RA.

Time window and exit rules: a sufficient treatment window (3–6 months) should be reserved to achieve clinically meaningful weight loss (typically threshold of ≥5%–10%) alongside IR improvement. Upon reaching targets, discontinuation and washout should be performed according to the prescribing label before initiating stimulation or embryo transfer.

Evidence calibration: current studies on improving ART outcomes are primarily small-scale and heterogeneous, suggesting “possible benefit” rather than a definitive guarantee (154, 193–196). Correcting the metabolic phenotype may lead to more favorable responsiveness and cumulative pregnancy outcomes in subsequent cycles, though prospective RCT validation is still required (170, 197).

Nutrition and anti-rebound: rapid weight loss warrants attention to nutritional adequacy, particularly in patients with restricted intake or gastrointestinal adverse effects. During discontinuation and washout, a structured plan to minimize weight regain and glycaemic deterioration is advisable–for example, reinforcing dietary/physical-activity targets and, where clinically indicated, using metformin as a maintenance option–so that metabolic stability is preserved before ART initiation.

*Scenario B: Ovulatory Dysfunction and Natural Conception (Moderate Risk, Recommended)*

Target Population: Patients with obesity and meaningful metabolic burden who have responded poorly to first-line ovulation induction (OI) with letrozole or clomiphene.

Strategic positioning: in this setting, GLP-1RAs are best positioned as adjunct metabolic optimization to improve the response to standard OI, rather than as substitutes for OI. The intent is to reduce metabolic load and IR first, then transition to conventional OI and timed intercourse (192, 193, 195).

Pathway key points: GLP-1RA therapy is used within a defined preconception optimization window. After pre-specified weight/metabolic targets are achieved, treatment is discontinued and the label-informed washout is completed before attempting conception or initiating an OI cycle. Available evidence supports improvements in weight and IR that may plausibly enhance HPO-axis responsiveness and shorten time to pregnancy; however, direct evidence for improvements in hard endpoints (particularly live birth) remains limited and should be communicated as such (17, 198, 199).

Core safety boundary: contraception is mandatory during the treatment period; “attempting pregnancy while on medication” does not align with current safety parameters.

*Scenario C: Lean or Non-metabolic Dominant PCOS (Not Recommended)*

General stance: current evidence does not support routine use of GLP-1RAs in PCOS patients with a BMI < 25 kg/m^2^ and no clear metabolic abnormalities. The primary conflict in patients is typically not rooted in the “metabolic foundation,” and blind use may incur costs such as gastrointestinal adverse reactions and the loss of lean body mass (190, 191, 200).

Suggested phrasing: preferred wording: “Not recommended outside strict indications (ideally within research protocols) and requires time-bounded use with explicit contraception and washout planning.” This avoids absolute prohibition while still signaling the expected standard of care.

*Scenario D: Unintended Pregnancy During GLP-1RA Therapy (Safety Management)*

Background: unintended conception may occur during GLP-1RA-assisted weight-loss treatment, particularly if contraception is inconsistent during dose escalation or periods of improved cycle regularity.

Decision: immediate discontinuation of the drug. Based on existing registry data (e.g., FDA and EMA data), the patient should be informed that while there is no clear evidence of teratogenicity, intensified prenatal monitoring is required. Blind termination of the pregnancy solely due to drug exposure is not recommended, thus avoiding unnecessary iatrogenic miscarriages.

### 8.3 Clinical decision matrix: stratification by metabolic phenotype and reproductive timeline

#### 8.3.1 Dimensions

Vertical Axis: High Risk: BMI ≥ 30 kg/m^2^ comorbid with IR, MASLD, impaired glucose tolerance (IGT), or a significant family history of cardiovascular disease. Low Risk: BMI < 30 kg/m^2^ with no clear metabolic abnormalities or overall low cardiometabolic risk.

Horizontal Axis: Immediate/Short-term: ≤1 year, or currently in the preparation phase for IVF/ICSI. Intermediate: Fertility plans within 1–3 years. Long-term/None: No intention for pregnancy within ≥3 years.

#### 8.3.2 Core recommendations

Based on these intersecting dimensions, we propose the following tiered recommendations (Table 1):

**TABLE 1:** Decision recommendation matrix for GLP-1RA use in PCOS infertility management, stratified by metabolic risk and fertility time window.

Patient phenotype	Reproductive timeline	Recommendation level	Key clinical operations
High metabolic risk (BMI ≥ 30, IR, MASLD)	Near-term (≤1 year) or preparing for ART	Strongly recommended	3–6 months intensive weight loss; washout after reaching target; sequential metformin to prevent rebound.
High metabolic risk (BMI ≥ 30)	Mid-to-long term (1–3 years)	Recommended	Targeted at long-term cardiometabolic protection; intensified LARC contraception; dynamic assessment of ovarian reserve.
Low metabolic risk (BMI < 30, no metabolic disorder)	Any time	Not routinely recommended	Prioritize lifestyle intervention or first-line OI protocols (e.g., letrozole).

This table provides clinicians with a structured basis for prescription based on baseline metabolic load and the urgency of fertility intentions. High metabolic risk is defined as BMI ≥ 30 kg/m^2^ with comorbid IR, MASLD, or IGT. Recommendation tiers: (i) Strongly Recommended: GLP-1RAs may be used as time-bounded metabolic optimization before OI or ART, aiming to improve metabolic readiness prior to stimulation and pregnancy planning. (ii) Recommended: prioritizes cardiometabolic risk reduction and longer-term health optimization in women with fertility plans that are not immediate. (iii) Not Routinely Recommended: emphasizes lifestyle intervention and first-line OI where appropriate, to avoid unnecessary exposure when metabolic burden is low and fertility urgency is high. Safety assumptions: all pathways assume effective contraception while on therapy and completion of product label–informed washout before conception attempts (e.g., semaglutide: discontinue at least 2 months before a planned pregnancy).

#### 8.3.3 Visualized clinical pathway

**FIGURE 3:**
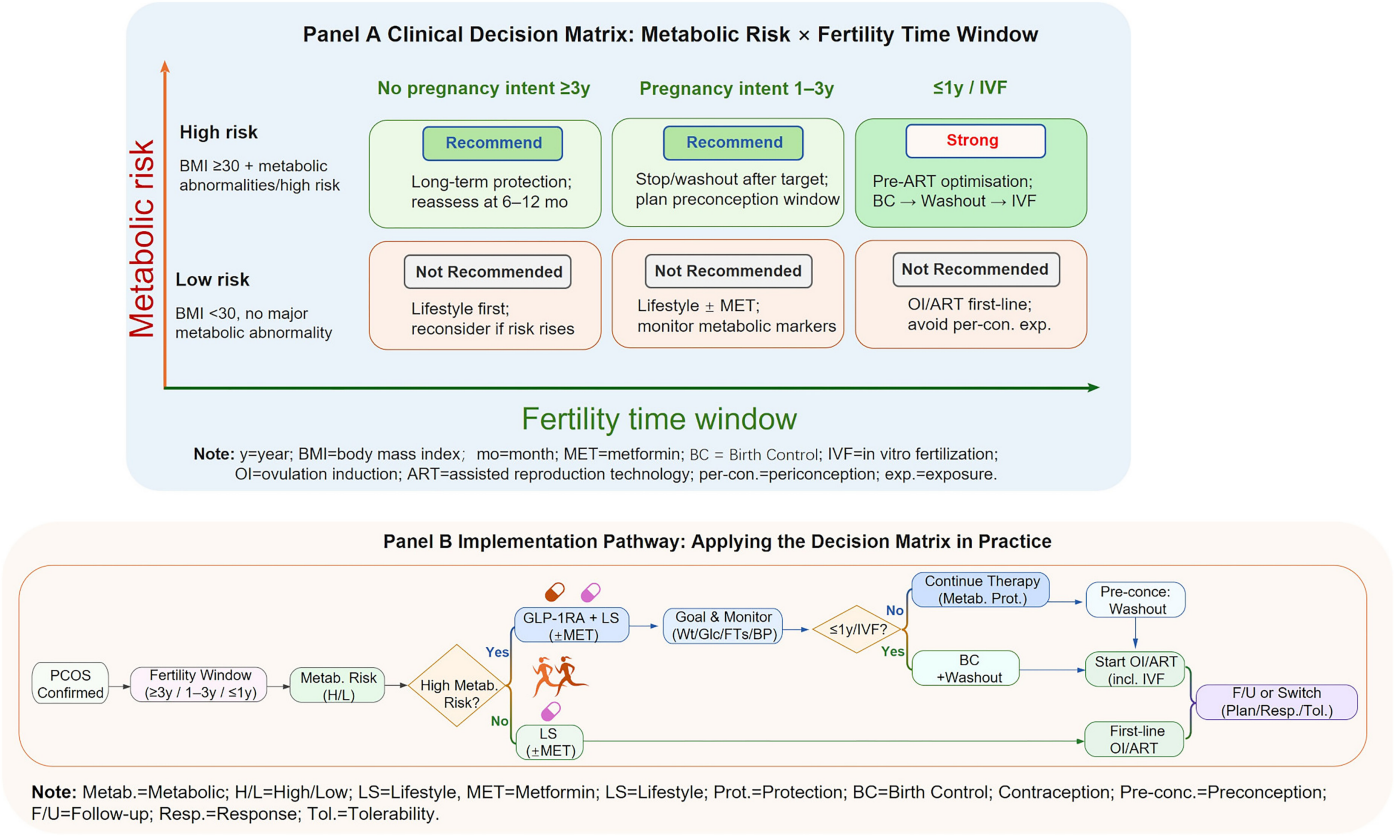
Clinical decision matrix and implementation pathway for GLP-1RA use in PCOS, stratified by metabolic risk and fertility time window. **(A)** Stratification by “Metabolic Risk (High/Low) × Reproductive Timeline,” presenting recommendation strengths and key implementation points. **(B)** Transformation of the matrix into a process: assess fertility intent and metabolic risk → (If applicable) Initiate GLP-1RA + Lifestyle (**±**MET**)** → Pre-emptive contraception and washout planning (per principles in Section “6 Periconception management and fertility planning: opportunities and risks”) → Transition to OI or ART → Follow-up and dynamic adjustment.

The matrix is operationalized in a flowchart (Figure 3). The pathway begins with confirmation of PCOS and clarification of reproductive intent, followed by stratification of metabolic burden (including BMI and key metabolic comorbidities). It then maps patients to the appropriate treatment tier, specifies the intended treatment window and monitoring targets, and–when pregnancy is planned–details discontinuation and washout timing before proceeding to timed intercourse, ovulation induction, or ART. This visual workflow is intended to support consistent counseling and documentation of key decision points in fertility-focused practice.

### 8.4 Health economics and access: important implementation questions, but no PCOS-ART-specific economic evidence yet

A structural tension exists between the clinical promise and the cost of GLP-1RAs. In the general obesity population, cost-effectiveness is highly sensitive to drug price, treatment duration, and reimbursement context, and published models have reached variable conclusions (201–203). However, no formal cost-effectiveness analysis was performed in the present review, and we did not identify a robust economic evaluation specifically addressing women with PCOS undergoing fertility treatment or ART pretreatment. Accordingly, the economic value of GLP-1RAs in this key reproductive scenario remains uncertain.

At present, the most defensible conclusion is implementation-oriented rather than economic: access, reimbursement, equity, and opportunity cost are likely to shape uptake more strongly than efficacy alone (204, 205). This is particularly relevant for fertility-focused use, where treatment duration is time-bounded, washout is mandatory, and downstream reproductive outcomes remain uncertain. Future PCOS-specific economic studies should model not only weight and metabolic effects, but also fertility timing, washout-related delay, ART utilization, and potential rebound-related costs.

## 9 Research gaps and future priorities

### 9.1 Reproductive-oriented RCTs: shifting from metabolic surrogates to hard endpoints

To date, most GLP-1RA trials in PCOS have been designed around metabolic efficacy (weight change and insulin-resistance/glycaemic indices) as the primary outcomes, with reproductive outcomes–ovulation, clinical pregnancy, and live birth–reported inconsistently and often as secondary or exploratory measures. Consequently, the existing evidence base is insufficient to support definitive recommendations in fertility management guidelines (195, 206, 207). The critical objective for the next phase of research is not to redundantly prove “weight loss efficacy,” but to determine whether and through which pathways these gains translate into benefits for “hard” reproductive endpoints.

Priority design elements: (i) Statistical Power and Follow-up. Trials must be powered for live birth (or cumulative live birth)–not merely metabolic surrogates–necessitating multi-center recruitment. Follow-up should span 12–24 months to capture post-discontinuation conception rates and metabolic trajectories (including weight rebound) after cessation. (ii) Pathway-Aligned Endpoints. Endpoints must be defined upfront according to the fertility pathway. Natural/OI: confirmed ovulation, clinical pregnancy, and cumulative live birth. ART: live birth (per transfer and cumulative), OHSS, and early loss. Crucially, denominators must be explicit (e.g., per started cycle *vs.* per transfer) to ensure comparability and prevent selective reporting. (iii) Mechanistic Biomarkers. To validate mechanisms beyond speculative claims of “oocyte quality,” protocols should embed quantitative biomarkers, such as follicular fluid metabolomics, embryo morphology, mitochondrial function, or endometrial molecular signatures. Where feasible, mediation analyses should distinguish direct reproductive effects from those driven solely by weight loss. (iv) Comparators and Timeline Standardization. Controls should mirror contemporary standard care (lifestyle ± metformin). Protocols must strictly specify the discontinuation-to-conception timeline (washout duration) to minimize time-related heterogeneity and ensure comparable exposure windows across study arms.

### 9.2 Periconception exposure and safety: quantifying risks within the “gray zone”

Periconception and gestational safety remain the weakest yet most restrictive links in the current evidence landscape. While existing systematic reviews and retrospective cohorts have not identified stable, potent teratogenic signals, the heterogeneity in exposure windows, drug types/dosages, and baseline metabolic risks–compounded by residual confounding–renders any “safe *vs.* unsafe” conclusion premature (44, 45, 154).

Future research should prioritize three keys: (i) Establishing Periconception Exposure Registries via Multidisciplinary Coordination: Prospective registration of GLP-1RA exposure cases from 3 months preconception through the 12th week of gestation is essential. Standardized records should include drug type, dosage, duration, interval between discontinuation and conception, and concomitant medications or contraceptive methods. (ii) Stratification and Long-term Follow-up of Maternal-Fetal Outcomes: Short-term outcomes (miscarriage, preterm birth, structural malformations, placental complications, fetal growth abnormalities) and mid-to-long-term outcomes (offspring growth trajectories, metabolic phenotypes) must be pre-defined within a standardized follow-up framework to assess potential “metabolic programming” risks. (iii) Enhancing Causal Inference Quality in the Absence of RCTs: Priority should be given to strategies such as target trial emulation, propensity score methods, negative controls, and sensitivity analyses. These approaches constrain confounding and selection bias during the design phase rather than relying on *post hoc* statistical corrections.

The ultimate goal is to transition from “theoretical risks based on animal data” to “communicable risk intervals based on population data,” providing a quantifiable basis for periconception risk counseling.

### 9.3 Bayesian and ML-driven DTRs: optimizing initiation, duration, and cessation

In fertility management, the pivotal questions regarding GLP-1RAs are often not about “efficacy” in isolation, but rather the optimal timing of initiation, treatment duration, cessation, and sequencing with OI/ART–classic Dynamic Treatment Regime (DTR) problems. Traditional static comparisons fail to capture the link between “strategy, timing, and outcome,” whereas methods such as Q-learning, A-learning, and tree-based reinforcement learning have demonstrated potential in identifying optimal strategies for chronic disease management (208–210).

High-priority methodological tasks include: (i) Utilizing real-world cohorts to evaluate the strategic effects of “initiation timing–regime duration–washout window” on long-term metabolic and reproductive outcomes. (ii) Integrating RCT evidence as informational priors within a Bayesian framework, fused with real-world data for dynamic updates, to output “strategy posteriors” rather than single-point effect estimates. (iii) Utilizing simulation and decision analysis to compare the long-term net benefits of divergent strategies (e.g., short-term intensive weight loss followed by discontinuation for conception *vs.* long-term maintenance until ART completion) to inform prospective trial designs and power calculations.

Boundary conditions must be clearly defined: data quality, missingness/attrition mechanisms, algorithmic interpretability, and fairness audits must be addressed *a priori*. Models should prioritize clinically interpretable structures (e.g., tree models, Bayesian networks, or explainable ensemble models) to facilitate integration into shared decision-making in the clinic.

### 9.4 Patient-centered outcomes: quantifying shared decision-making as evidence

Polycystic Ovary Syndrome face complex trade-offs between body weight, aesthetic distress, fertility goals, psychological burden, and long-term cardiometabolic risks. Previous research suggests that patient expectations are primarily focused on weight loss, aesthetic improvement, and opportunities for conception, while long-term cardiovascular risks receive relatively less attention (211–213). Without the quantification and contextualization of these preferences, guideline-level “weak recommendations” or “shared decision-making” remain difficult to implement.

Future work should also address three implementation-oriented priorities. First, quantify patient preferences. Discrete choice experiments can formalize the trade-offs patients and clinicians routinely make–between expected weight loss, chances of conception, adverse effects, out-of-pocket costs, and the possibility of post-discontinuation regain–so that Evidence-to-Decision processes can incorporate empirically derived weights rather than implicit assumptions. Second, build decision-support outputs that communicate uncertainty. Bayesian posteriors and phenotype-stratified models should be translated into visual probability displays (e.g., threshold probability plots and risk–benefit trade-off charts) that enable comparison of clinically relevant pathways (metabolic optimization first versus immediate OI/ART), instead of presenting single-point predictions that overstate precision. Third, ensure equity and accessibility. Preference weights and willingness-to-pay (or affordable cost thresholds) should be validated across diverse socioeconomic and healthcare-access contexts, so that decision tools do not inadvertently encode the values and constraints of resource-abundant settings.

## 10 Conclusions and clinical take-home messages

### 10.1 Main findings: certainties amidst uncertainties

Synthesizing the current evidence, the information architecture regarding GLP-1RAs in PCOS fertility management exhibits a characteristic gradient: “robust metabolic evidence, hypothesis-generating reproductive findings, and a gray zone of safety.”

Robust metabolic benefit: in overweight or obese PCOS phenotypes, evidence for the improvement of metabolic endpoints–including body weight, central adiposity, and IR–is consistently positive, facilitating the establishment of actionable therapeutic goals (e.g., reaching clinically significant weight-loss thresholds and optimizing IR markers). However, it must be noted that direct trial evidence for cardiovascular “event endpoints” and long-term outcomes specifically within the PCOS population remains scarce. Therefore, clinical claims should focus on improving cardiometabolic risk factors and metabolic substrates rather than prematurely extrapolating “MACE reduction” data from general obesity populations.

Reproductive gains as hypothesis-generating findings: while favorable exploratory findings are observed in outcomes such as menstrual regularity, androgenic markers, and natural conception/TTP, these findings are generally limited by small sample sizes, restricted follow-up windows, and a lack of gold-standard ovulatory measurements. In particular, the paucity of data regarding live birth and cumulative live birth rates necessitates that these outcomes be viewed as “potential benefits requiring validation” rather than confirmed reproductive gains at this stage.

Periconception safety within a precautionary gray zone: an “absence of a stable teratogenic pattern” is not synonymous with “proven safety.” In the absence of robust human exposure-outcome evidence, periconception management must adhere to a defensive principle: continuous effective contraception combined with adherence to drug-specific discontinuation and washout protocols remains the mandatory safety boundary.

Role repositioning: the appropriate clinical role of GLP-1RAs is not to replace ovulation induction or ART, but to serve as a periconception metabolic optimization tool. By attenuating IR and lipotoxicity and enhancing metabolic stability, these agents provide a more controllable physiological substrate for subsequent reproductive interventions.

### 10.2 Communicating benefits, limits, and safety boundaries

Within the current evidence and regulatory framework, GLP-1RA management should be proceduralized using explicit entry points, target goals, and exit rules to minimize uncertainty and risk spillover:

The “triple-question” pre-prescription assessment: before prescribing, clinicians should systematically address three questions: (i) What is the patient’s metabolic risk tier (BMI/IR, MASLD, glycemic status, etc.)? (ii) What is the fertility time window (≤1 year/preparing for IVF; 1–3 years; or ≥3 years)? (iii) Is the current contraceptive method reliable and sustainable throughout the treatment period? This assessment ensures that contraception and discontinuation are discussed before initiation.

Precision screening: candidates most likely to benefit are those with higher metabolic risk and a near-term fertility timeline. In practice, this often includes women with obesity (e.g., BMI ≥ 30 kg/m^2^)–or those close to this threshold with clinically meaningful IR and/or metabolic comorbidities such as MASLD or impaired glucose tolerance–who have a defined plan for conception or ART within the next year. By contrast, routine initiation is generally not indicated for lean PCOS or for patients with low cardiometabolic risk, except in selected circumstances (e.g., specific metabolic indications) or within research protocols.

Target-driven exit rules: transition the clinical mindset from “treating for a trial period” to “exiting upon target achievement.” For instance, a 3–6 months metabolic optimization window should be pre-set to achieve a clinically meaningful weight-loss threshold and improve IR markers; a mandatory washout phase follows this before transitioning to OI or ART. The priority is achieving a “metabolic stability window” rather than pursuing the lowest possible weight.

Managing the “Rebound Window” as a High-Risk Node: the interval between drug discontinuation, washout, and the initiation of conception or IVF is the most vulnerable phase. “Anti-rebound” strategies should be integrated into the clinical pathway, including intensified lifestyle maintenance and, where appropriate, sequential metformin to maintain metabolic stability. Weight trajectories and metabolic markers should serve as follow-up triggers to prevent acute fluctuations during sensitive reproductive windows.

Communicating benefits, limits, and safety boundaries: GLP-1RAs should be communicated as tools for metabolic optimization rather than as fertility-enhancing guarantees. At the same time, improved ovulatory function during treatment may increase the risk of unintended conception if contraception is inconsistent. The key periconception safety boundaries remain explicit contraception during treatment, drug-specific discontinuation, and completion of the recommended washout period before conception attempts. Shared decision-making should therefore integrate patient priorities regarding weight reduction, treatment tolerability, cost, timing of conception, and readiness for OI or ART, rather than relying on body weight alone as the sole marker of success.

### 10.3 Perspectives: from a single drug to an adaptive precision-care paradigm

Future progress in this field will depend not only on more effective agents, but also on higher-quality reproductive evidence and better integration of metabolic, reproductive, and safety endpoints into clinical pathway design. Key priorities include: (i) reproductive-oriented RCTs with live birth or cumulative live birth as primary endpoints and standardized washout templates; (ii) periconception exposure registries and stronger causal-inference strategies to better characterize maternal-fetal risk; and (iii) phenotype-stratified, clinically interpretable decision-support tools that can help identify which patients are most likely to benefit from time-bounded metabolic optimization before conception or ART.

Under the combined influence of stronger evidence, clearer safety boundaries, and patient values, GLP-1RAs may help shift PCOS management from isolated interventions toward a more integrated, life-course-oriented care framework.
