# Supplementary material for: Balancing metabolic optimization and reproductive safety in Polycystic Ovary Syndrome: a Bayesian-informed framework for GLP-1 receptor agonists
Source: Front Nutr. 2026 Jun 2;13:1809416. doi: 10.3389/fnut.2026.1809416 (PMC13269364; doi:10.3389/fnut.2026.1809416)
Supplement: Supplementary file 1 [file Data_Sheet_1.docx]

# **Supplementary Materials**

Appendix 1. Proof-of-Concept Bayesian Network Meta-analysis: Dataset and Inclusion Rules

Figure S1. Adapted PRISMA flow diagram for the proof-of-concept Bayesian NMA subset

Table S1. RCTs in PCOS included ≥1 GLP-1RA arm and reported body-weight change

Appendix 2. Bayesian Consistency NMA

Appendix 3. Computation, Convergence, and Model Fit

Figure S2. Trace plots for the RE consistency Bayesian NMA

Figure S3. Trace plots for the FE consistency Bayesian NMA

Table S2. MCMC convergence diagnostics for the Bayesian NMA

Table S3. Model fit and complexity: DIC and residual deviance

Table S4. Arm-level residual deviance contributions from the RE consistency model

Appendix 4. Definitions and Interpretation of Main Outputs

Appendix 5. Random-Effects (Primary) Results: Heterogeneity and ROPE

Table S5. RE main analysis: ROPE-based posterior probabilities vs metformin

Appendix 6. Fixed-Effect Sensitivity Analysis and Robustness

Appendix 7. Structured Evidence-Certainty Summary Across Clinical Domains

Table S6. Structured evidence-certainty summary across outcome domains relevant to fertility-centered PCOS care

Appendix 8. Molecule-specific Periconception Safety, Pharmacokinetic, and Washout Considerations

Table S7. Molecule-specific periconception safety, pharmacokinetic, and washout considerations for GLP-1 receptor agonists

Supplementary Data Files

Data S1. Fixed-effect ROPE outputs versus metformin

Data S2. PoC Bayesian NMA input dataset

Data S3. Treatment name harmonization map

Data S4. Bayesian NMA statistical analysis code

**Appendix 1 | Proof-of-Concept Bayesian Network Meta-analysis: Dataset and Inclusion Rules**

This appendix documents the data source, trial selection, outcome harmonization, and inclusion rules used for the proof-of-concept (PoC) Bayesian network meta-analysis (NMA) of body-weight change in women with polycystic ovary syndrome (PCOS).

**1.1 Data Acquisition and Construction of the Evidence Base**

We systematically searched PubMed (MEDLINE) for randomized controlled trials (RCTs) evaluating glucagon-like peptide-1 receptor agonists (GLP-1RAs) in women with polycystic ovary syndrome (PCOS). The search strategy comprised four modules: PCOS population terms, GLP-1RA terms, an RCT filter, and exclusion of animal-only studies. The core PubMed search string was as follows:

(("Polycystic Ovary Syndrome"[Mesh] OR "polycystic ovary syndrome"[tiab] OR PCOS[tiab] OR "polycystic ovarian syndrome"[tiab])

AND

("Glucagon-Like Peptide 1 Receptor Agonists"[Mesh] OR "GLP-1 receptor agonist*"[tiab] OR GLP-1RA*[tiab] OR liraglutide[tiab] OR semaglutide[tiab] OR exenatide[tiab] OR dulaglutide[tiab] OR tirzepatide[tiab] OR lixisenatide[tiab] OR albiglutide[tiab] OR efpeglenatide[tiab])

AND

(randomized controlled trial[pt] OR controlled clinical trial[pt] OR random*[tiab] OR placebo[tiab] OR trial[tiab]))

NOT (animals[mh] NOT humans[mh])

For the PoC RCT evidence base, studies were eligible if they: (i) enrolled women diagnosed with PCOS; (ii) included at least one GLP-1RA-containing intervention arm; (iii) used a randomized comparative design; and (iv) reported extractable body-weight change data. We excluded duplicate reports of the same trial, studies without extractable weight-change outcomes, studies with ineligible comparators for the connected network, and studies outside the prespecified 12–16-week primary window for the main NMA. For the broader narrative synthesis, relevant observational studies, reviews, and clinical guidance were retained where they informed reproductive outcomes, periconception exposure, washout, or implementation issues.

A total of 88 records were retrieved. After title/abstract screening, 25 reports underwent full-text assessment, of which 23 provided extractable weight outcomes. One report comparing PCOS versus non-PCOS controls (rather than GLP-1RA vs non-GLP-1RA) was excluded, yielding a weight evidence base of 22 reports. Duplicate publications addressing the same trial from different perspectives were merged, resulting in 17 unique RCTs. For the proof-of-concept Bayesian network meta-analysis (NMA) focused on weight change, we first excluded one trial due to lack of network connectivity (16 candidate RCTs), and then prespecified a short-term primary time window of 12–16 weeks to mitigate follow-up-related heterogeneity. The primary NMA therefore included 11 RCTs (8 nodes, 25 arms; **Figure S1**).

.
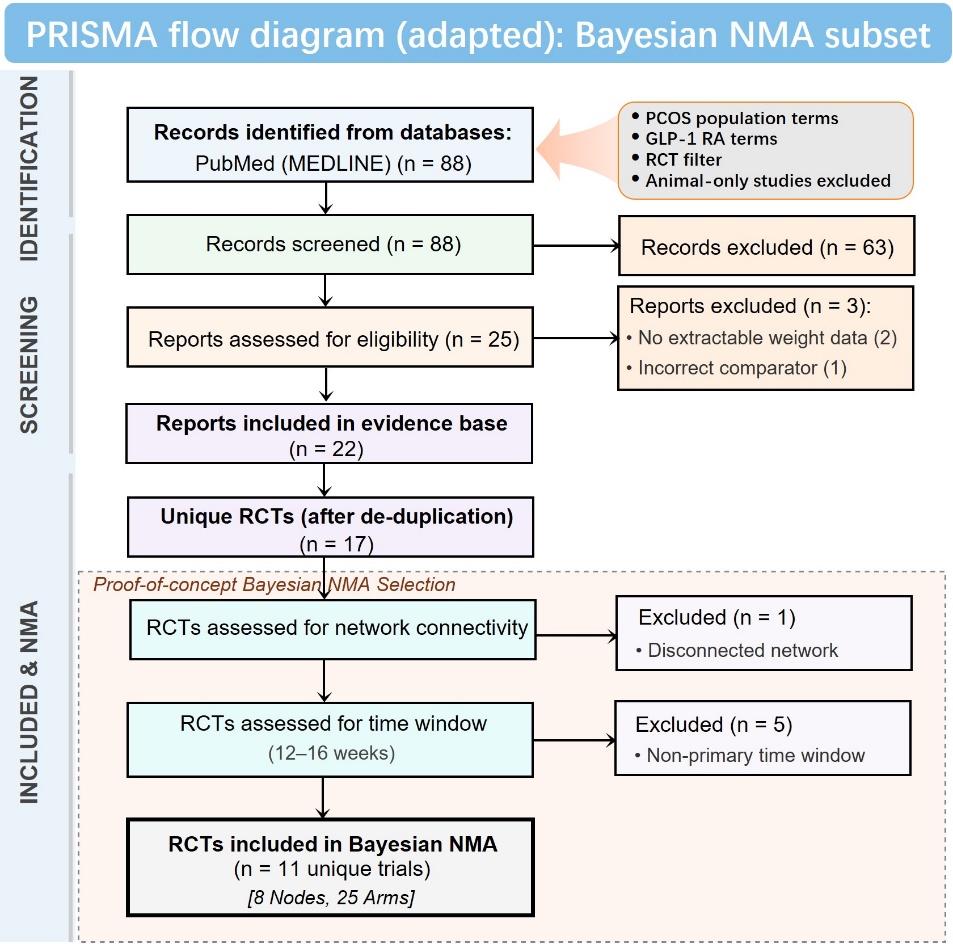


**Figure S1** Adapted PRISMA flow diagram for the proof-of-concept (PoC) Bayesian NMA subset

**Legend:** The search strategy combined PCOS population terms, GLP-1RA terms, and an RCT filter, with animal-only studies excluded. PubMed (MEDLINE) identified 88 records; 63 were excluded during screening. Twenty-five reports were assessed for eligibility, and 3 were excluded due to non-extractable weight data (n=2) or an ineligible comparator (n=1), leaving 22 reports in the evidence base. After de-duplication, 17 unique RCTs were retained. For the PoC Bayesian NMA dataset, 1 RCT was excluded due to a disconnected network, and 5 were excluded for not meeting the prespecified main time window (12–16 weeks, inclusive). Ultimately, 11 RCTs were included in the Bayesian NMA (8 treatment nodes, 25 arms).

Trials contributing to the weight evidence base are summarized in **Table S1**, with all comparator arms listed to preserve network transparency and connectivity.

**Table S1** RCTs in PCOS included ≥1 GLP-1RA arm and reported body-weight change (all comparator arms listed)

| study | Country | Duration | node | dose | n | mean_cha  nge_kg | sd_cha  nge_kg | baseline_  mean_kg |
| --- | --- | --- | --- | --- | --- | --- | --- | --- |
| Li R2022 | China | 12 | exenatide | 10-20μg/d | 72 | -5.21 | 3.94 | 73.72 |
| Li R2022 | China | 12 | metformin | 1000 mg BID | 75 | -3.55 | 2.13 | 72.37 |
| Liao2024 | China | 12 | cpa-ee+metformin | 2mg/d+35μg/d | 30 | -1.85 | 3.25 | 71.14 |
| Liao2024 | China | 12 | liraglutide+metformin | 1.2-1.8mg/d | 30 | -7.4 | 5.91 | 74.56 |
| Ma2021 | China | 12 | exenatide+metformin | 2 mg QW | 19 | -3.8 | 2.4 | 82.34 |
| Ma2021 | China | 12 | metformin | MET 500 TID | 21 | -2.1 | 3 | 79.1 |
| Xuesong D2025 | China | 12 | exenatide+metformin | 2mg QW +1500mg/d | 35 | -2.69 | 5.7 | 85.14 |
| Xuesong D2025 | China | 12 | metformin | 1500mg/d | 31 | -2.38 | 4.18 | 79.38 |
| Jensterle 2015 | Slovenia | 12 | liraglutide | 1.2mg/d | 14 | -3.1 | 3.5 | 102.8 |
| Jensterle 2015 | Slovenia | 12 | metformin | 1000mg, BID | 13 | -0.2 | 1.83 | 108.3 |
| Jensterle 2015 | Slovenia | 12 | roflumilast | 0.5mg/d | 14 | -2.1 | 2 | 111.1 |
| Tao2021 | USA | 12 | exenatide | 10-20μg/d | 50 | -5.45 | 4.34 | 80 |
| Tao2021 | USA | 12 | exenatide+metformin | 10-20μg/d+1500-2000 mg/d | 50 | -6.52 | 3.7 | 83.17 |
| Tao2021 | USA | 12 | metformin | 1500-2000 mg | 50 | -4 | 3.06 | 80 |
| Jensterle2014 | Slovenia | 12 | liraglutide | 1.2 mg/d | 11 | -3.8 | 3.7 | 108.9 |
| Jensterle2014 | Slovenia | 12 | liraglutide+metformin | 1.2 mg/d+1000 mg BID | 11 | -6.5 | 2.8 | 105.5 |
| Jensterle2014 | Slovenia | 12 | metformin | 1000 mg BID | 14 | -1.2 | 1.4 | 103.2 |
| Salamun V2018 | Slovenia | 12 | liraglutide+metformin | 1.2 mg/d+1000 mg BID | 13 | -7.51 | 3.89 | 106.6 |
| Salamun V2018 | Slovenia | 12 | metformin | 1000 mg BID | 14 | -6.99 | 6.02 | 99.6 |
| Xing2022 | China | 12 | liraglutide+metformin | 1.2 mg/d | 27 | -9.13 | 3.71 | 79.09 |
| Xing2022 | China | 12 | metformin | 1000mg BID | 25 | -5.06 | 5.43 | 76.5 |
| Jensterle2016 | Slovenia | 12 | liraglutide | 1.2mg/d | 21 | -3.8 | 3.5 | 102.6 |
| Jensterle2016 | Slovenia | 12 | liraglutide+metformin | 1.2mg/d+1000mg BID | 22 | -6.2 | 2.4 | 105.8 |
| Chen2025 | China | 16 | metformin | 1000mg BID | 40 | -2.25 | 4.27 | 70.55 |
| Chen2025 | China | 16 | semaglutide+metformin | 1.0mg QW | 40 | -6.09 | 3.34 | 73.06 |
| Jensterle2025 | Slovenia | 16 | placebo |  | 15 | 2.2 | 5.5 | 100.7 |
| Jensterle2025 | Slovenia | 16 | semaglutide | 1.0 mg QW | 15 | -5.6 | 4.5 | 100.3 |
| Elkind-Hirsch KE2021 | USA | 24 | dapagliflozin | 10mg/d | 17 | -1.4 | 7.6 | 104 |
| Elkind-Hirsch KE2021 | USA | 24 | dapagliflozin+metformin | 10mg/d+2000mg | 19 | -1.8 | 7.65 | 103 |
| Elkind-Hirsch KE2021 | USA | 24 | exenatide | 2mg QW | 20 | -4.1 | 7.84 | 104.5 |
| Elkind-Hirsch KE2021 | USA | 24 | exenatide+dapagliflozin | 2mg QW+10mg/d | 20 | -6 | 7.36 | 104 |
| Elkind-Hirsch KE2021 | USA | 24 | phentermine/topiramate | Phen 7.5mg/d+ TPM ER 46mg/d | 16 | -9 | 7.26 | 106 |
| Zhang2023 | China | 24 | calorie-restricted diet |  | 33 | -5.44 | 1.17 | 78.84 |
| Zhang2023 | China | 24 | dulaglutide+diet | 1.5 mg QW | 35 | -5.42 | 1.12 | 77.62 |
| Frøssing2018 | Denmark | 26 | liraglutide | 1.8 mg/d | 44 | -5.2 | 4.64 | 94.2 |
| Frøssing2018 | Denmark | 26 | placebo |  | 21 | 0.2 | 4.12 | 91.3 |
| Elkind-Hirsch 2022 | USA | 32 | liraglutide | 3.0mg/d | 44 | -6.3 | 5.37 | 111 |
| Elkind-Hirsch 2022 | USA | 32 | placebo |  | 23 | -1.1 | 6.71 | 119 |
| Elkind-Hirsch2008 | USA | 24 | exenatide | 5-10ug BID | 14 | -3.2 | 1.38 | 110.5 |
| Elkind-Hirsch2008 | USA | 24 | exenatide+metformin | 5-10ug BID+1000 mg BID | 14 | -6 | 7 | 112 |
| Elkind-Hirsch2008 | USA | 24 | metformin | 1000 mg BID | 14 | -1.6 | 2.81 | 113.4 |

**Note:** This table summarizes arm-level data extracted from all eligible randomized controlled trials (RCTs) included in the review. Because each trial contained at least one GLP-1RA arm, non-GLP-1RA interventions (e.g., calorie-restricted diet, dapagliflozin, phentermine/topiramate, roflumilast, placebo) are also listed when they served as comparator arms within the same trials, to fully represent network structure and connectivity. Duration indicates the intervention period (weeks). mean_change_kg denotes the mean change in body weight from baseline to the reported follow-up end point (kg), with negative values indicating weight loss; sd_change_kg is the standard deviation of the change; baseline_mean_kg is the mean baseline body weight (kg); n is the sample size per arm (as reported); node indicates the treatment node used in the network meta-analysis (NMA); dose is reported as described in the original publications. For the PoC Bayesian NMA, weight change was re-expressed as weight-loss magnitude (mean_loss = − mean_change_kg, where positive values indicate greater weight loss). When multiple time points were available, the 12–16-week window (inclusive) was prioritized for the main analysis

**Abbreviations:** GLP-1RA, glucagon-like peptide-1 receptor agonist; MET, metformin; CPA-EE, cyproterone acetate–ethinylestradiol; QD, once daily; BID, twice daily; TID, three times daily; QW, once weekly; SD, standard deviation; SE, standard error; NMA, network meta-analysis; PoC, proof-of-concept.

**1.2 Outcome and Time Window**

This PoC NMA analysed body-weight change (kg) only. The prespecified primary short-term window was 12–16 weeks (inclusive).

To ensure a consistent direction for ranking and probabilistic interpretation, “weight change” (negative values indicating weight loss) was converted to magnitude of weight loss (positive values indicating greater loss) as:

$$\text{mean\_loss}=-\text{ mean}$$

Accordingly, model-based relative effects $\left( \Delta\right)$**greater than 0** indicate **more weight loss than metformin** (the reference).

**1.3 Network Connectivity**

Because the 12–16-week data contained disconnected comparisons in the full network, this PoC analysis restricted inference to the largest connected component to meet NMA identifiability requirements. Subnetwork A comprised 11 trials, 8 treatments (nodes), and 25 study arms. The treatment nodes included in Subnetwork A were: metformin, liraglutide, liraglutide+metformin, exenatide, exenatide+metformin, roflumilast, cpa/EE+metformin, semaglutide+metformin.

Comparisons that were disconnected from Subnetwork A (e.g., a 16-week comparison forming an isolated two-node network) were not included in this PoC NMA.

Consistency and local inconsistency assessment. Because the primary connected network was sparse and included only limited closed loops, formal node-splitting/local inconsistency analyses were not considered sufficiently informative to serve as a primary decision tool in this proof-of-concept NMA. Accordingly, consistency was judged pragmatically through network geometry, clinical transitivity, comparison of random-effects and fixed-effect results, and deviance-based model fit diagnostics.

**Appendix 2 | Bayesian Consistency NMA**

This appendix provides the full Bayesian technical specification underlying the proof-of-concept NMA, including model structure, priors, computation, convergence diagnostics, model fit, ROPE definitions, and sensitivity analyses, which are summarized only briefly in the main text.

**2.1 Likelihood and Link Function (continuous outcome)**

For each study $i$and treatment arm $k$, the observed mean weight-loss magnitude $y_{ik}$was assumed normally distributed:

$$y_{ik}\sim\mathcal{N}(\mu_{ik},\mathrm{SE}_{ik}^{2})$$

where $\text{SE}_{ik}$was derived from the reported SD and sample size $n$(when SD was available, $\text{SE}=\text{SD}/\sqrt{n}$). An identity link was used. Study-specific baselines were represented by trial intercepts:

$$\mu_{ik}=u_{i}+\delta_{t(i,k)}$$

where $t(i,k)$denotes the treatment in arm $k$of study $i$. **Metformin** was set as the reference treatment, with $\delta_{\text{metformin}}=0$.

**2.2 Fixed-effect and Random-effects Models**

- **Fixed-effect (FE) model:** assumes a common true relative effect $\delta_{t}$shared across studies.
- **Random-effects (RE) model (primary analysis):** allows study-specific true effects to vary around the overall effect, captured by the between-study SD $\tau$:

$$\delta_{i,t}\sim\mathcal{N}(\delta_{t},\tau^{2})$$

**2.3 Prior Distributions**

This PoC analysis used weakly informative priors (consistent with common/default settings in **gemtc**) to avoid undue prior influence:

- **Trial intercepts:**

$$u_{i}\sim\mathcal{N}(0,{10}^{4})$$

- **Treatment effects (non-reference):**

$$\delta_{t}\sim\mathcal{N}(0,{10}^{4}),t\neq\text{metformin}$$

- **Heterogeneity:**

$$\tau\sim\text{Uniform}(0,5)\text{ kg}$$

**Appendix 3 | Computation, Convergence, and Model Fit**

**3.1 MCMC Settings**

Posterior sampling was performed via Markov chain Monte Carlo (MCMC) using multiple chains with over-dispersed initial values. Standard burn-in and sampling iterations were applied to ensure stable posterior estimation (details correspond to the implementation settings reported in the accompanying code/output).

**3.2 Convergence Assessment**

Convergence was evaluated using **trace plots** and the **Gelman–Rubin potential scale reduction factor (PSRF)**. Key parameters showed adequate mixing and stationarity; PSRF values were approximately 1, indicating satisfactory convergence（**Table S2**, **Figure S2–S3**）.


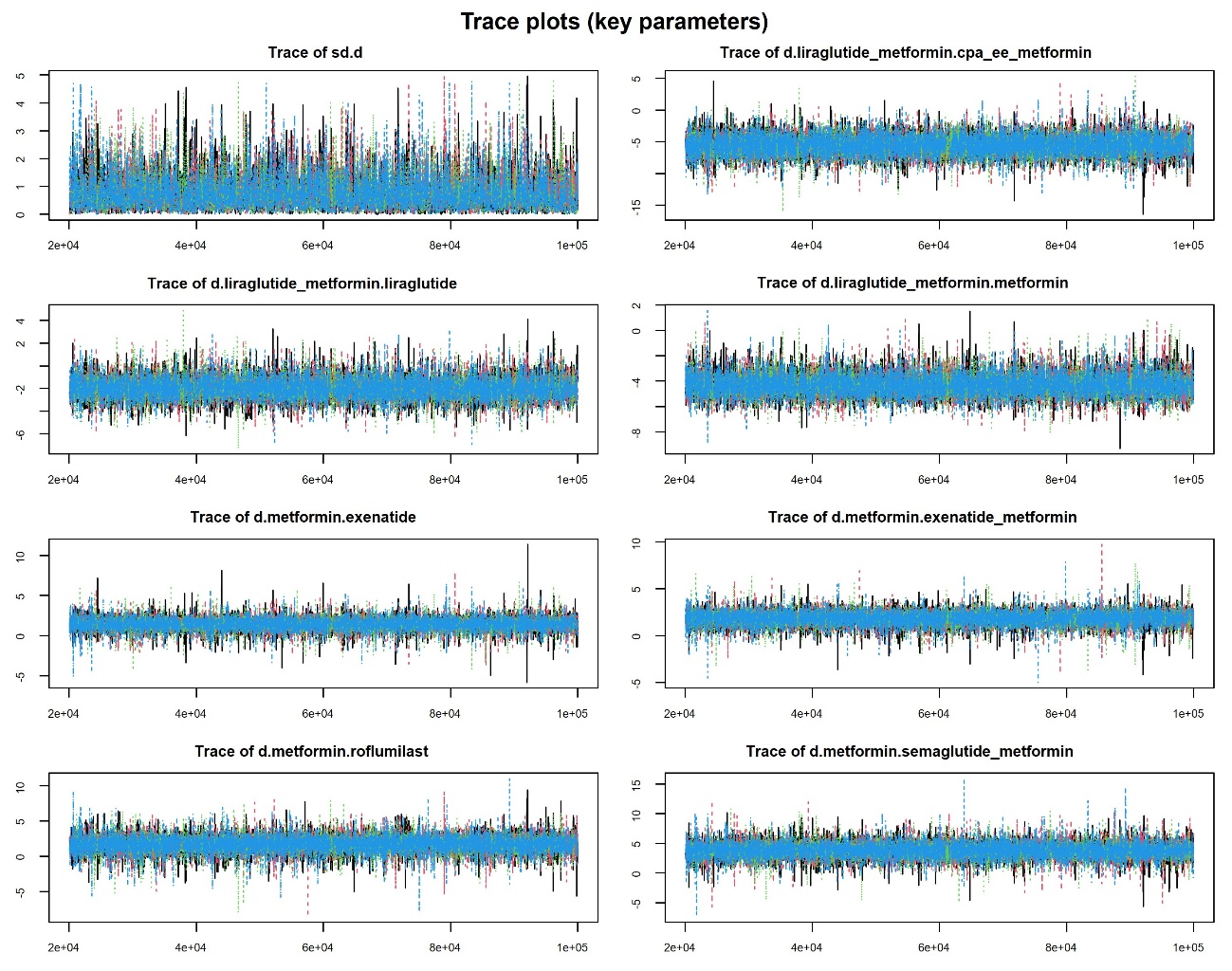


**Figure S2** Trace plots for the RE consistency Bayesian NMA (key parameters)

**Legend:** Trace plots are shown for the random-effects consistency Bayesian NMA to assess MCMC convergence and mixing. After burn-in, trajectories exhibit stable “hairy caterpillar” patterns without discernible drift, with good overlap across chains, indicating adequate convergence. The figure includes the heterogeneity parameter (sd.d) and selected treatment effect parameters (d.*). Full trace plots for all parameters and PSRF (R-hat) diagnostics are provided in the Appendix/Supplementary files. Key parameters displayed: sd.d, d.liraglutide_metformin.cpa_ee_metformin, d.liraglutide_metformin.liraglutide, d.liraglutide_metformin.metformin, d.metformin.exenatide, d.metformin.exenatide_metformin, d.metformin.roflumilast, d.metformin.semaglutide_metformin


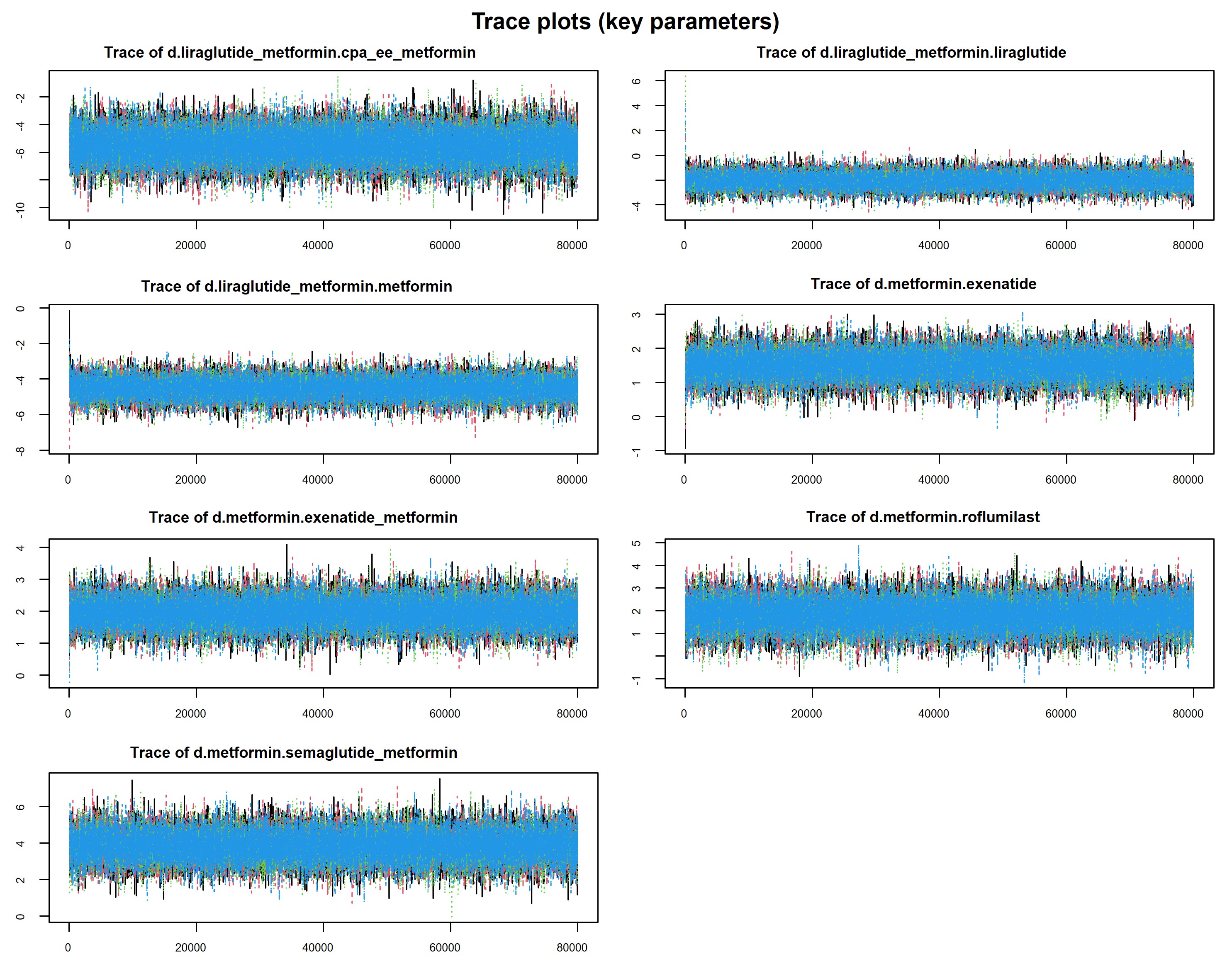


**Figure S3**| Trace plots for the FE consistency Bayesian NMA (key parameters)

**Legend:** Trace plots for the fixed-effects consistency Bayesian NMA are shown to confirm convergence in the sensitivity analysis. After burn-in, parameter trajectories fluctuate around stable levels with good between-chain mixing, supporting adequate convergence. Selected treatment effect parameters (d.*) are displayed. Full trace plots for all parameters and PSRF (R-hat) diagnostics are available in the Appendix/Supplementary files. Key parameters displayed: d.liraglutide_metformin.cpa_ee_metformin, d.liraglutide_metformin.liraglutide, d.liraglutide_metformin.metformin, d.metformin.exenatide, d.metformin.exenatide_metformin, d.metformin.roflumilast, d.metformin.semaglutide_metformin。

Convergence was additionally supported by PSRF values ≈1.00 for all monitored parameters (**Table S2**).

**Table S2** MCMC convergence diagnostics for the Bayesian NMA (12–16 weeks): Gelman–Rubin PSRF (R-hat)

| Param | Rhat | Rhat_upper | Model |
| --- | --- | --- | --- |
| d.liraglutide_metformin.cpa_ee_metformin | 1.000337816 | 1.0009593287149956 | RE |
| d.liraglutide_metformin.liraglutide | 1.000009212 | 1.0000243473149186 | RE |
| d.liraglutide_metformin.metformin | 1.000221622 | 1.0005703585179644 | RE |
| d.metformin.exenatide | 1.000068509 | 1.0001774011696156 | RE |
| d.metformin.exenatide_metformin | 1.000299215 | 1.00065922000648 | RE |
| d.metformin.roflumilast | 1.000224304 | 1.0005653974427 | RE |
| d.metformin.semaglutide_metformin | 1.00029062 | 1.0007272302619885 | RE |
| sd.d | 1.000356581 | 1.0006683495949333 | RE |
| Multivariate PSRF | 1.000658073 | NA | RE |
| d.liraglutide_metformin.cpa_ee_metformin | 0.999997985 | 1.0000347218080414 | FE |
| d.liraglutide_metformin.liraglutide | 1.000132993 | 1.0003531589785923 | FE |
| d.liraglutide_metformin.metformin | 1.000032257 | 1.0001786687101784 | FE |
| d.metformin.exenatide | 1.000161147 | 1.0005546834224595 | FE |
| d.metformin.exenatide_metformin | 0.999967207 | 1.000012763814442 | FE |
| d.metformin.roflumilast | 1.000069325 | 1.0002148401940887 | FE |
| d.metformin.semaglutide_metformin | 0.999993773 | 1.000028646588447 | FE |
| Multivariate PSRF | 1.000201258 | NA | FE |

**Legend:** This table reports Gelman–Rubin potential scale reduction factors (PSRF; R-hat) and the corresponding upper bounds (Upper 95% CI) for key parameters from the consistency random-effects model (RE; primary analysis) and the consistency fixed-effects model (FE; sensitivity analysis). R-hat values were close to 1.00 for all monitored parameters, with upper bounds near 1, indicating adequate between-chain convergence. Multivariate PSRF values were also close to 1, supporting overall convergence. Parameters d.* denote relative treatment effects and sd.d denotes the between-study heterogeneity (SD) parameter in the RE model.

**3.3 Model Fit**

We computed Deviance Information Criterion (DIC) and residual deviance (posterior mean deviance; Dbar) as supportive indicators of model fit and complexity (**Table S3**). In connected subnetwork A (12–16 weeks), residual deviance was close to the number of data points for both models (RE: Dbar = 25.37; FE: Dbar = 26.27; data points = 25), suggesting an adequate overall fit. As expected, the RE model had a larger effective number of parameters than the FE model (pD = 20.35 *vs*. 18.02), reflecting increased complexity when allowing for between-study heterogeneity. The FE model showed a slightly lower DIC than the RE model (44.29 *vs.* 45.72; ΔDIC≈1.44), indicating only marginal preference by this criterion. Given the small DIC difference and the proof-of-concept nature of this analysis, we retained the RE model for the primary analysis to acknowledge plausible heterogeneity, and used the FE model as a sensitivity analysis to assess robustness.

**Table S3** | Model fit and complexity: DIC and residual deviance for the RE and FE consistency Bayesian NMA

| model | DIC | pD | residual_deviance | data_points | resid_per_datapoint |
| --- | --- | --- | --- | --- | --- |
| RE | 45.72472 | 20.34974127 | 25.37497872 | 25 | 1.014999149 |
| FE | 44.28626623 | 18.0163456 | 26.26992062 | 25 | 1.050796825 |

**Footnote:** This table reports DIC, the effective number of parameters (pD), and residual deviance (posterior mean deviance; Dbar) for the RE (primary) and FE (sensitivity) consistency models. The residual deviance is benchmarked against the number of data points as a coarse check of model fit (Dbar close to the number of data points generally suggests adequate overall fit). DIC, which penalizes model complexity, is used as a supportive criterion for comparing RE versus FE and is not treated as the sole basis for model selection in this proof-of-concept analysis.

**3.4 Residual Deviance Contributions and Influential Points**

To screen for potential misfit and influential study arms, we extracted arm-level contributions to the residual deviance from the random-effects (RE) consistency model (**Table S4**). This diagnostic reflects how well each arm aligns with the model-predicted mean response; markedly larger contributions may indicate locally influential or outlying arms and warrant targeted verification against the extracted trial data.

Across connected subnetwork A (12–16 weeks), most arm-level deviance contributions were of a comparable magnitude (approximately 0.5–1.3), suggesting broadly balanced fit. Notably, **Salamun_V2018** showed relatively larger contributions (~1.73 and ~2.59), indicating greater tension with the network fit. Given its small sample size (n = 13–14) and similar mean weight loss between arms (7.51 *vs* 6.99 kg) alongside sizeable variability (SD 3.89 and 6.02), the larger contributions may plausibly reflect random variation, large within-arm variance, or local incompatibility with the network-average effects. We therefore prioritised this trial for targeted data checks (e.g., consistency of units/time window, arm definitions, and extracted summary statistics). Entries of **NA** in the third arm (V_3_) arise from two-arm trials stored in a fixed-width arm matrix and do not represent missing data.

**Table S4** Arm-level residual deviance contributions from the RE consistency model: screening for misfit and influential arms

| study | arm | dev_contrib |
| --- | --- | --- |
| Chen2025 | V1 | 0.987564490946732 |
| Chen2025 | V2 | 1.0031770295984137 |
| Chen2025 | V3 | NA |
| Jensterle 2015 | V1 | 0.7459144753565288 |
| Jensterle 2015 | V2 | 0.9187995437728239 |
| Jensterle 2015 | V3 | 0.9900086251319387 |
| Jensterle2014 | V1 | 0.5011393516821802 |
| Jensterle2014 | V2 | 1.0449482424426006 |
| Jensterle2014 | V3 | 0.9849341381134603 |
| Jensterle2016 | V1 | 0.8509435078120806 |
| Jensterle2016 | V2 | 0.9301734757978115 |
| Jensterle2016 | V3 | NA |
| Li_R2022 | V1 | 0.8670262133754444 |
| Li_R2022 | V2 | 0.9604636854785709 |
| Li_R2022 | V3 | NA |
| Liao2024 | V1 | 1.0144153530467872 |
| Liao2024 | V2 | 1.0007948785214245 |
| Liao2024 | V3 | NA |
| Ma2021 | V1 | 0.8221626603677775 |
| Ma2021 | V2 | 0.7584419662147075 |
| Ma2021 | V3 | NA |
| Salamun_V2018 | V1 | 1.7285450828758098 |
| Salamun_V2018 | V2 | 2.592192039116845 |
| Salamun_V2018 | V3 | NA |
| Tao2021 | V1 | 0.8069324221921585 |
| Tao2021 | V2 | 1.0878350088098414 |
| Tao2021 | V3 | 0.8616372291233882 |
| Xing2022 | V1 | 0.8471671277934093 |
| Xing2022 | V2 | 0.6201564122499404 |
| Xing2022 | V3 | NA |
| Xuesong D2025 | V1 | 1.2859795821799205 |
| Xuesong D2025 | V2 | 1.1636261826397036 |
| Xuesong D2025 | V3 | NA |

**Note:** This table reports arm-level contributions to the residual deviance for connected subnetwork A (12–16 weeks) under the RE consistency Bayesian NMA model. Larger values indicate greater deviation between an arm’s observed summary outcome and the model-implied prediction, which may suggest local misfit or an influential arm. Columns V1–V3 denote fixed arm slots within each study; NA values in V3 occur for two-arm trials due to structural padding and do not indicate missing data. These diagnostics are presented as supportive information to guide targeted data verification and sensitivity checks.

**Appendix 4 | Definitions and Interpretation of Main Outputs (Relative Effects, Ranking, ROPE)**

**4.1 Relative Effects**

For each treatment, we report the posterior mean and 95% credible interval (CrI) **relative to metformin**. Because the outcome is **mean_loss**, $\Delta>0$indicates greater weight loss than metformin.

**4.2 Ranking and SUCRA**

Ranking probabilities $P(\text{rank}=r)$and SUCRA values were computed from posterior samples. In this PoC analysis, FE and RE produced identical SUCRA ordering (Spearman $=1.00$; maximum rank difference $=0$), suggesting that rankings were not sensitive to the FE/RE specification. As expected, RE yielded **more dispersed** ranking probabilities (more conservative), reflecting explicit modelling of between-study heterogeneity.

**4.3 ROPE (clinically negligible difference) and Posterior Probabilities**

To enhance clinical interpretability, we defined a ROPE interval $\left[ -\delta,+\delta\right]$and calculated:

- **PROPE**：$P(\mid\Delta\mid\leq\delta)$: probability that the difference is clinically negligible
- $\boldsymbol{P}_{\mathbf{dir}}$**：**$P(\Delta>0)$ : probability favoring the treatment direction
- $\boldsymbol{P}_{\boldsymbol{>}\boldsymbol{\delta}}$**：**$P(\Delta>\delta)$ : probability that the effect exceeds the clinical threshold

The primary threshold was $\delta=1.0$kg, with a sensitivity analysis at $\delta=0.5$kg.
**Note:** $\Delta$posterior mean and 95%CrI do **not** change with $\delta$; only ROPE-derived probabilities ($P_{\text{ROPE}}$, $P_{>\delta}$) depend on the chosen threshold.

**Appendix 5 | Random-Effects (Primary) Results: Heterogeneity and ROPE (vs metformin)**

**5.1 Heterogeneity Parameter（**$\boldsymbol{\tau}$**）**

Under the RE model, the posterior mean of between-study heterogeneity was $\tau=0.674$kg (95%CrI 0.020–2.221 kg). This parameter is reported for heterogeneity assessment and is not interpreted as a treatment node.

**5.2 RE Main Analysis**

Under the RE primary analysis ($\delta=1.0$ kg), combined regimens (liraglutide+metformin; semaglutide+metformin) showed high posterior probabilities of exceeding a 1-kg advantage over metformin, with liraglutide and exenatide+metformin also likely to surpass this threshold. Exenatide and roflumilast showed favorable directions of effect but with greater uncertainty (CrI crossing 0). Results were directionally consistent when the threshold was tightened to $\delta=0.5$kg (**Table S5**).

**Table S5** RE main analysis: ROPE-based posterior probabilities vs metformin (δ = 1.0 and 0.5 kg)

| Treatment node (vs metformin) | Δ Mean (95%CrI), kg | δ=1.0: PROPE | δ=1.0: P(Δ>1) | δ=0.5: PROPE | δ=0.5: P(Δ>0.5) |
| --- | --- | --- | --- | --- | --- |
| liraglutide + metformin | 4.34 (2.59–5.86) | 0.0017 | 0.998 | 0.0009 | 0.999 |
| semaglutide + metformin | 3.84 (1.39–6.28) | 0.0133 | 0.985 | 0.0056 | 0.991 |
| liraglutide | 2.36 (0.61–3.97) | 0.0556 | 0.943 | 0.0166 | 0.980 |
| exenatide + metformin | 1.84 (0.36–3.21) | 0.0957 | 0.902 | 0.0279 | 0.967 |
| roflumilast | 1.73 (−0.44–3.81) | 0.2026 | 0.785 | 0.0830 | 0.894 |
| exenatide | 1.43 (−0.14–2.87) | 0.2284 | 0.766 | 0.0729 | 0.914 |
| CPA/EE + metformin | −1.22 (−4.71–2.05) | 0.3679 | 0.086 | 0.1884 | 0.147 |

**Note:** *Δ* denotes additional weight loss versus metformin (on the mean_loss scale; *Δ* > 0 indicates greater weight loss). CrI denotes the 95% credible interval. PROPE is defined as $P(\mid\Delta\mid\leq\delta)$, i.e., the posterior probability that the effect lies within the ROPE. $P(\Delta>\delta)$is the posterior probability that the effect exceeds the prespecified clinical threshold. The posterior mean and CrI for Δ are derived from the same random-effects model and therefore do not vary across ROPE thresholds; only the ROPE-based probabilities depend on $\delta$.

**Abbreviations**: CrI, credible interval; PROPE, posterior probability within ROPE; CPA/EE, cyproterone acetate–ethinylestradiol.

**Appendix 6 | Fixed-Effect Sensitivity Analysis and Robustness**

The FE model yielded the same SUCRA ordering as the RE model, but with more concentrated (less conservative) ranking probabilities, as FE does not explicitly model between-study heterogeneity. Given the posterior for $\tau$suggested non-trivial heterogeneity, the RE model was prespecified as the primary analysis, with FE used to confirm robustness of conclusions. Key FE ROPE outputs are provided in **ROPE_FE_vs_metformin_delta1.csv**.

**Appendix 7 | Structured Evidence-Certainty Summary Across Clinical Domains**

**Table S6** Structured evidence-certainty summary across outcome domains relevant to fertility-centered PCOS care

| Outcome domain | Main evidence base | Overall direction of evidence | Key limitations affecting certainty | Certainty / interpretive level |
| --- | --- | --- | --- | --- |
| **Weight loss** | Multiple randomized trials in women with PCOS, including monotherapy and combination regimens; PoC Bayesian NMA restricted to the connected 12–16-week network | Consistent short-term benefit of GLP-1RA-containing regimens versus metformin, placebo, or some active comparators, especially for combination regimens | Trial sizes are often modest; treatment duration varies; some comparisons are open-label; network is sparse for some nodes; long-term maintenance is insufficiently characterized in PCOS | **Moderate-to-high certainty for short-term metabolic benefit** |
| **Insulin resistance / glycemic-metabolic improvement** | Randomized trials reporting HOMA-IR, fasting glucose, OGTT-derived measures, HbA1c, waist circumference, visceral/ectopic fat markers | Direction generally favors GLP-1RA-containing regimens, especially in overweight/obese or insulin-resistant phenotypes | Endpoints are heterogeneous across trials; not all studies report the same metabolic metrics; some improvements may be partly mediated by weight loss rather than directly separable from it | **Moderate certainty** |
| **Ovulation / menstrual recovery / endocrine improvement** | Randomized trials and short-term follow-up studies reporting menstrual cyclicity, ovulation, LH/FAI/SHBG and related endocrine markers | Suggestive improvement in menstrual cyclicity, some ovulation-related measures, and selected endocrine parameters in several studies | Definitions and denominators vary; endocrine endpoints are inconsistently reported; follow-up is short; concomitant metformin and comparator structure differ substantially | **Low-to-moderate certainty; hypothesis-generating** |
| **Clinical pregnancy / natural conception** | Small trials and post-treatment follow-up studies; published meta-analytic summaries from heterogeneous trial sets | Possible increase in natural conception after preconception metabolic optimization | Pregnancy endpoints are inconsistently defined; conception windows and washout timing vary; no confident adjustment for weight-loss mediation, confounding, or treatment discontinuation structure; event counts remain limited | **Low certainty; exploratory** |
| **IVF / ART outcomes, including live birth-related translation** | Very limited number of small studies, including pilot IVF pretreatment evidence | Signals of possible benefit for selected intermediate ART-related outcomes in some settings | Evidence base is sparse; sample sizes are very small; open-label designs are common; live birth and cumulative ART outcomes are underreported; no confirmatory synthesis is currently possible | **Low certainty; exploratory and non-confirmatory** |
| **Periconception exposure safety** | Pregnancy registries, observational exposure cohorts, case series, and narrative syntheses; limited direct PCOS-specific exposure data | No stable teratogenic signal has been identified to date | Exposure cohorts are small and heterogeneous; rare outcomes are underpowered; timing and duration of exposure vary; confounding by maternal obesity/diabetes and indication cannot be fully excluded | **Low certainty; insufficient for a safety conclusion** |

**Note:** This table presents a structured narrative certainty summary derived from the evidence synthesized in the main text and Supplementary Materials, including the RCT evidence base, proof-of-concept Bayesian NMA subset, and the broader reproductive and periconception safety literature reviewed in this article. It is intended as an interpretive summary rather than a formal de novo GRADE evidence profile, and therefore individual references are not listed within each cell. Representative supporting references are cited in the corresponding sections of the main text.

**Appendix 8 | Molecule-specific Periconception Safety, Pharmacokinetic, and Washout Considerations**

**Table S7** Molecule-specific periconception safety, pharmacokinetic, and washout considerations for GLP-1 receptor agonists

| Agent | Approximate half-life | Label-informed discontinuation/washout before planned conception | Available human pregnancy-exposure evidence (representative scale and type of evidence) | Reported outcomes | Certainty note |
| --- | --- | --- | --- | --- | --- |
| **Semaglutide** | ~1 week | At least 2 months before planned pregnancy | Human pregnancy-exposure evidence is available mainly from class-level early-pregnancy GLP-1RA cohorts and systematic reviews; semaglutide-specific exposed pregnancy counts remain limited | No stable pattern of major structural malformations has been identified to date; however, exposure cohorts remain heterogeneous and underpowered for rare but clinically important harms | **Low certainty** for human periconception safety; suitable for a precautionary, exposure-avoidance approach rather than a safety conclusion |
| **Liraglutide** | ~13 hours | If pregnancy is desired or occurs, discontinue; no explicit fixed preconception interval is stated in the current product labeling | Human data remain limited and are derived largely from case reports, pharmacovigilance reports, registry-based data, and class-level GLP-1RA observational cohorts/reviews | No stable teratogenic pattern has been established in humans, but the evidence base is too small and heterogeneous to exclude uncommon risks with confidence | **Low certainty**; shorter half-life than semaglutide, but still not supported by sufficient human safety data for planned periconception exposure |
| **Exenatide** | Immediate-release formulation: ~2.4 hours | No explicit preconception interval is stated in current product labeling; planned exposure should be avoided, and use in pregnancy is generally not preferred unless benefit clearly justifies risk | Human pregnancy-exposure data are very sparse; evidence comes mainly from isolated reports and small numbers embedded within broader GLP-1RA class-level datasets | No stable malformation signal has been identified, but molecule-specific human evidence is especially limited; interpretation is constrained by very small exposed numbers | **Very low certainty**; practical preconception planning should remain formulation-specific |
| **Dulaglutide** | ~5 days | No explicit fixed preconception interval is stated in the current product labeling; planned exposure should be avoided and use during pregnancy is not routinely supported | Human pregnancy-exposure evidence is very limited, consisting mainly of isolated reports and small numbers within class-level observational datasets/reviews | No stable human teratogenic pattern has been established, but available data are insufficient for meaningful reassurance, especially for rare outcomes | **Very low-to-low certainty**; long half-life supports a cautious preconception discontinuation strategy even though labels do not specify a fixed interval |

**Note:** Approximate half-life and label-informed discontinuation/washout entries were derived from current official product labeling for semaglutide, liraglutide, exenatide, and dulaglutide. Human pregnancy-exposure evidence is summarized here as the representative scale and type of available data rather than as additive cumulative totals, because observational cohorts, case reports, and later reviews may include overlapping primary datasets. Accordingly, this table is intended as an interpretive clinical summary rather than a formal de novo pooled exposure analysis. Because direct molecule-specific exposed pregnancy counts remain small for most agents, the absence of a stable teratogenic signal should not be interpreted as proof of safety.

**Supplementary Data Files (DOI:** **10.17632/8ng9r59hgb.1)**

**Data S1 | Fixed-effect ROPE outputs versus metformin (δ = 1.0 kg).** ROPE_FE_vs_metformin_delta1.csv

**Data S2 | PoC Bayesian NMA input dataset (12–16 weeks, Subnetwork A).** Arm-level long-format dataset used for *gemtc/JAGS* modelling (study, treatment/node, mean_loss, SD, sampleSize, followup_weeks). NMA_ab_12_16_subnetA_gemtc.csv

**Data S3 | Treatment name harmonization map.** Mapping from trial-reported treatment labels (treatment) to analysis-ready node names (treatment_safe) used in the PoC Bayesian NMA. treatment_name_map.csv

**Data S4 | Bayesian NMA statistical analysis code.** R scripts and supporting files used to reproduce the PoC analyses (compressed as a single ZIP archive). BAIYES-NMA-R-CODE.rar
